# What We Need to Know about Liposomes as Drug Nanocarriers: An Updated Review

**DOI:** 10.34172/apb.2023.009

**Published:** 2022-04-04

**Authors:** Hanieh Abbasi, Maryam Kouchak, Zohreh Mirveis, Fatemeh Hajipour, Mohsen Khodarahmi, Nadereh Rahbar, Somayeh Handali

**Affiliations:** ^1^Department of Medicinal Chemistry, School of Pharmacy, Ahvaz Jundishapur University of Medical Sciences, Ahvaz, Iran.; ^2^Nanotechnology Research Center, Ahvaz Jundishapur University of Medical Sciences, Ahvaz, Iran.; ^3^Department of Pharmaceutics, School of Pharmacy, Ahvaz Jundishapur University of Medical Sciences, Ahvaz, Iran.; ^4^Medical Biomaterials Research Center (MBRC), Tehran University of Medical Sciences, Tehran, Iran.

**Keywords:** Liposome, Liposome synthesis, Liposome targeting methods, Liposome loading methods, Liposome applications

## Abstract

Liposomes have been attracted considerable attention as phospholipid spherical vesicles, over the past 40 years. These lipid vesicles are valued in biomedical application due to their ability to carry both hydrophobic and hydrophilic agents, high biocompatibility and biodegradability. Various methods have been used for the synthesis of liposomes, so far and numerous modifications have been performed to introduce liposomes with different characteristics like surface charge, size, number of their layers, and length of circulation in biological fluids. This article provides an overview of the significant advances in synthesis of liposomes via active or passive drug loading methods, as well as describes some strategies developed to fabricate their targeted formulations to overcome limitations of the "first-generation" liposomes.

## Introduction

 Nowadays, the use of nanomaterials as a system for drug-delivery has been widely considered, specially, in cancer therapy.^1^ It has been proved that materials in nanoscale (˂ 200 nm) can prolong the circulation time in body as well as entering the cells *via* endocytosis; consequently, cause intracellular absorption.^2,3^ Different nanomaterials such as micelles,^4^ dendrimers,^5,6^ superparamagnetic iron oxide nanoparticles (SPIONs),^7^ mesoporous silica nanoparticles,^8^ gold nanoparticles (GNPs),^9^ quantum dots,^10^ carbon nanotubes,^11^ and liposomes have been used in drug delivery systems.^12^ Among them liposomes are the most common nanocarriers due to their inherent advantages such as high biocompatibility, low immunogenicity, cell-like membrane, low toxicity, and ability to protect drugs from hydrolysis and prolong their biological half-life. They are able to encapsulate either hydrophobic or hydrophilic molecules and control the drug release.^3,13,14^ Besides, many efforts have been made in developing of smart drug carriers that deliver their cargo in response to an external or internal trigger. In this regard, liposomes are recognized as one of the most successful drug delivery systems.^15,16^

**Figure 1:**
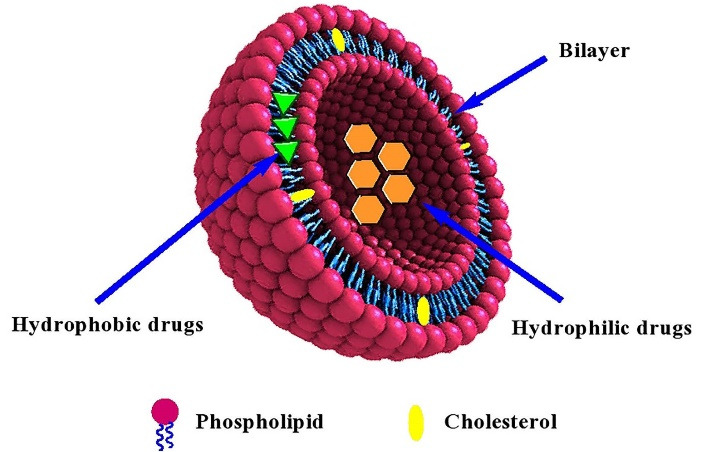
Schematic presentation of structure of a typical liposome loaded with hydrophilic and hydrophobic drugs

 In general, liposomes are sphere-shaped microscopic vesicles with the hydrophilic portion completely enclosed by one or more phospholipid bilayers (Figure 1).^17^ Due to the amphiphilic nature of phospholipids, they favor to assemble as closed bilayer structures in such a way that minimize the confrontation between aqueous and hydrophobic domains. So, the lowest free energy state and the maximum stability to self-assembled structures are achieved. Besides, the hydrodynamic and other destabilizing forces can cause the fragmentation of the bilayer to form the smaller liposomes.^18,19^*In vivo* and *in vitro* stability of liposomes are controlled by their physical and chemical characteristics such as lipid composition, size, charge, number of lamellae and surface modifications.^20^ Up to now, numerous researches related to liposomes have been performed owing to their importance in the nanomedicine field. Loaded drugs on liposomes can include a wide range of anti-cancer drugs, antibiotics, small interfering RNAs (siRNA), antisense oligonucleotides, and bacterial plasmids carrying therapeutic genes.^21^ Similarity of phospholipids to cell membrane facilitates passage of liposome through some membrane barriers for distribution in tissues and removal from the elimination organs. Besides, modification of liposomes with various ligands and polymers improves drug uptake and increases circulation time of drug in the blood.^12,22^ After the clinical approval of PEGylated liposomal doxorubicin (Doxil^®^) as the first nanodrug by US FDA in 1995, 19 liposomal formulations have been clinically approved for the treatment of various diseases. Nevertheless, there are major concerns about their stability, controlled and predictable pharmacokinetics and pharmacodynamics as well as reproducible production in large scale that needs improvement.^14,20,23^ One of the challenges in application of liposomes in clinical use is the interaction of liposome constituents with the immune system. Liposome components can induce antibody production which leads to reduction of their efficacy.^24^ In addition, the lack of established techniques for large-scale production of liposomes, and suitable models that exactly imitate tumor heterogeneity, are limitations for clinical development of liposomes.^25^

 Several review articles have been published describing liposomal structures, preparation methods and their application.^12,15,17,19,24-31^ The present review, besides the general aspects liposomes, focuses on the significant advances in their synthesis *via* active or passive drug loading methods, as well as describing some strategies developed to fabricate new-generation liposomes with target-specificity and stimuli-sensitivity.

## Structural units of liposomes

 In general, the structure of liposomes consists of two parts; phospholipid and cholesterol. Phospholipids are the major component of liposomal structure and cholesterol improves their stability. The hydrophilic head of these fats is a phosphate group that is joined to hydrophobic components by a water-soluble molecule like glycerol and can be natural or synthetic.^18,22^ A list of different types of phospholipids is presented in Table 1. Choosing the proper phospholipid for achieving the desired therapeutic goals is essential.^32-34^ Cholesterol incorporates within phospholipid bilayer because it cannot form liposomes alone. It is essential for the consolidation of bilayers, increasing the packaging of phospholipid molecules, controlling drug retention, and reducing the permeability of the bilayers.^17,32,35,36^

**Table 1:** Various types of phospholipids used in the preparation of liposomes

**Chemical name**	**Abbrev.**	**Formula**	**Source**	**Ref.**
Phosphatidylcholine	PC	C_42_H_82_NO_8_P	Egg yolk, soybeans	^37^
Phosphatidylethanolamine	PE	C_7_H_12_NO_8_PR_2_	Chocolate, soybean milk	^38^
1,2-Distearoyl-sn-glycero-3-phosphoethanolamine	DSPE	C_41_H_82_NO_8_P	Synthetic	^39^
Dimyristoyl phosphatidylcholine	DMPC	C_36_H_72_NO_8_P	synthetic	^29^
Dimyristoyl phosphatidylglycerol	DMPG	C_34_H_67_O_10_P	synthetic	^29^
Dipalmitoylphosphatidylcholine	DPPC	C40H80NO8P	cell membranes, pulmonary surfactant	^29^
Dioleoyphosphatidyl choline	DOPC	C_44_H_84_NO_8_P	synthetic	^29^
dipalmitoyl phosphatidyl glycerol	DPPG	C_38_H_75_O_10_P	mitochondrial membranes, pulmonary surfactant	^29^
2,3-dioleyloxy-N-[2(sperminecarbox amido)ethyl]-N,N-dimethyl-1-propanammonium trifluoroacetate	DOSPA	C_56_H_111_F_3_N_6_O_5_	synthetic	^29^
1,2-bis(oleoyloxy)-3-(trim ethylammonio)propane	DOTAP	C_10_H_25_N_3_	synthetic	^29^
1,2-dimystyloxypropyl-3-dimethyl hydroxyethyl ammonium bromide	DMRE	C_20_H_29_BrN_2_	synthetic	^29^
3β[N-(N',N'-dimethylaminoethane)-carbomoyl] cholesterol	DC-CHOL	C_32_H_56_N_2_O_2_	synthetic	^29^
Dioctadecylamino-glycyl-spermine	DOGS	C_10_H_26_N_4_	As polycation in eukaryotic cells	^29^
Phosphatidylinositol	PI	C_47_H_83_O_13_P	Endoplasmic reticulum	^40^
Phosphatidylserine	PS	C_13_H_24_NO_10_P	Soy, white beans, egg yolks, chicken liver, beef liver	^41^
Phosphatidic acid	PA	C_39_H_77_O_8_P	Cabbage and radish leaves, *Mallotus japonicas*	^42^
Phosphatidylglycerol	PG	C_40_H_77_O_10_P	Live amniotic fluid surfactant	^43^
Cardiolipin	CL	C_81_H_158_O_17_P_2_	Mammalian and plant cells, inner mitochondrial membrane	^44^

## Characteristics of liposomes

 The performance of liposomes depends on their size, number of layers, shape, and surface charge. Therefore, estimating and characterizing these properties is essential for clinical application as well as in determining their half-life.^19^ These colloidal vesicles have different number of layers are classified based on size and number of bilayers (Figure S1). They can be unilamellar (UV) or multilayer. UVs are also classified into four subgroups of small (SUVs), medium (MUVs), large (LUVs), and giant (GUVs) vesicles according to their size. The multilayer liposomes are divided to oligolamellar (OLVs), multilamellar (MLVs), and multivesicular (MVVs) vesicles.^45,46^ The zeta potential is the electrostatic charge of the particle surface that prevents the proximity and aggregation of particles.^47^ Zeta potential can provide perception about circulation times, stability, circulation times, and biocompatibility of nanoparticles.^48^ Moreover, Zeta potential is important factor in the initial adsorption of liposome onto the surface of cells.^49^

## Synthesis methods of liposomes

 Up to now, many methods have been reported for the production of liposomes which can be divided to conventional and novel techniques. Conventional strategies include thin-film hydration (Figure S2),^50^ reverse phase evaporation (Figure S3),^26^ ethanol injection (Figure S4),^51^ ether injection (Figure S5, Supplementary file 1),^52^ electro-formation,^53^ and detergent depletion methods.^54^ These methods are easy to implement and do not require complicated equipment; however, scale-up for industrial manufacture and scale-down for point-of-care applications are challenging issues of them.^19,55^ In addition, limitations in process control, poor reproducibility, and inefficient use of materials and reagents are other significant problems.^56^ For overcoming these problems, some *new methods* have been developed for preparation of liposome.

###  Microfluidic methods

 Microfluidic techniques refer to the strategies in which the procedures are performed in a small volume, typically in sub-millimeter scales and low Reynolds Numbers. By exploiting these microfluidic techniques the laboratory procedures can be performed in planar chips or other small devices result in reducing cost of chemical and biological experimentation.^56,57^ A number of microfluidic methods have been developed called modified Electro-formation,^58^ lipid hydration,^59^ micro hydrodynamic focusing,^60^ Pulsed jetting,^61^ double emulsion templates,^62^ lipid coated ice droplet hydration,^63^ transient membrane ejection^56,64^ modified droplet emulsion transfer^65^ either as modification of macro-scale techniques or as completely novel methods.

###  Supercritical fluid (SCF) based methods 

 Some new methods exploit SCF which is increasingly replacing organic solvents due to its ability for efficient separation and purification. There are several strategies for liposome preparation using SCF method. In a technique, a compressed mixture of the lipids, SCF and organic co-solvent is injected into the aqueous phase, and sprayed into water to form liposomes (injection method).

 Whereas, in another approach, the compressed phase composed of lipid, SCF as well as aqueous phase is sprayed into air through a nozzle (decompression method). The size of the obtained vesicles is related to the rate of depressurization. It has been claimed that through these methods sterile, solvent free and pharmaceutical grade liposomes having a narrow particle size distribution can be produced. The incorporation of aqueous phase is the major difference between these approaches.^19,54^ In another method, supercritical reverse phase evaporation (scRPE), a mixture of lipid, organic co-solvent and compressed gas are put in a stirred, variable volume cell above the lipid phase transition temperature, and then an aqueous solution is slowly introduced to the cell. The liposomes are formed upon the pressure is reduced by the release of the compressed gas. The principle of the scRPE method is similar to the decompression method. However, in this method the depressurization occurs by the release of the dense gas from a variable volume cell.^54,66^ In another method called supercritical anti-solvent precipitation, the phospholipid dissolved in an organic co-solvent is sprayed into the SCF as an anti-solvent, resulting in formation of micronized particles. The size of the particles depends on the droplet size of the spray and the concentration of the lipid in the co-solvent. After hydration of the particles in an aqueous buffer the liposomes are formed. It was reported that increase in the pressure of the system or the SCF/co-solvent ratio causes the reduction in the fraction of small liposomes in the system.^67,68^ It has been claimed that the scaling-up of the SCF methods can be implemented with less problems.^19^

###  Other new methods

 In the method called “freeze-drying double emulsions”, preparation of liposomes is accomplished by the lyophilization of double emulsions (W1/O/W2) containing disaccharides as lyoprotectants in both the inner and outer aqueous phase, by a two-step emulsification procedure at room temperature.^69^ “Membrane contactor” is a modified ethanol injection method in which phospholipid solution in alcohol was extruded through a membrane contactor into an aqueous solution and the liposomes are formed.^70^ In the method “hydration of deposited phospholipids on nanostructures” phospholipids are deposited on amphiphilic electrospun nanofibres composed of polyvinylpyrrolidone and soybean lecithin. The liposomes self-assembled upon addition of the nanofibers into water.^71^ In the other method namely “Curvature-tuning”, the phenomenon of spontaneous vesiculation and theory of curvature have been taken into consideration in solvent-free liposome preparation procedure. In this method, rapid pH change followed by a defined period of equilibration is exploited for the preparation of stable, monodisperse, and unilamellar liposomes. Further, by direct addition of the lipids into an aqueous buffer, there is no need to first preparation of MLVs suspension. The size, shape, and dispersity of the liposomes are affected by some critical factors such as time interval of pH increase, time of equilibration, temperature, and type of lipid.^19,72,73^

###  Large-scale techniques for liposome production

 Application of liposomal formulation in industrial scale has two challenging issues including; poor capability of transferring from academic bench to highly regulated technology and stability of liposomes.^74,75^ Ethanol injection method is one of the interesting methods for scaling-up production of liposomes owing to reproducibility, fast implementation, and simplicity. Moreover, this technique did not cause oxidation and degradation of lipids. It has been reported that by use of this method, 0.5 to 12 kg of liposomes can be obtained from batches.^76^ Microfluidic isanother effective reproducible method for scale up of liposomes. This method has a high potential to achieve more control over the physical properties of the end product, especially in terms of size distribution, lamellarity, and high encapsulation efficiency.^75,77,78^

## Modifications to conventional liposomes

 Vesicles with simple structure including; cholesterol and phospholipid are named conventional liposomes or “first-generation liposomes”. These liposomes have some drawbacks like fast release of drug, rapid elimination from the blood, capture by the mononuclear phagocyte system, and low entrapment efficiency of water–soluble drugs.^45,79^ To overcome the mentioned deficiencies some new strategies have been developed in liposome preparations and novel generations of these vesicles have been emerged.^30,80^

###  Fusogenic liposomes (FLs)

 Conventional liposomes are usually taken up into cells by phagocytosis or endocytosis and the main part of their content such as macromolecules might be degraded before reaching the cytoplasm.^79,81^ The induction of membrane fusion between liposomes and the cell membrane can overcome to this problem. FLs are nanocarriers which may fuse with biological membranes, thereby increasing drug contact and delivery into cells. FLs are composed of lipids, such as dioleoyl-phosphatidylethanolamine (DOPE) and cholesterylhemisuccinate (CHEMS), which cause increased fluidity in the lipid bilayer and can destabilize biological membranes.^82^ Due to their composition, the bilayer structure of FLs is efficiently fused with the cellular plasma membrane of cell to deliver the content of liposomes into the cytoplasm without degradation.^79,83^

 One of the most interesting types of FLs is virosomes. These FLs are prepared by incorporation of conventional liposomes-based phospholipid with UV-disabled Sendai virus. The presence of the Sendai virus allows liposomes to rapidly and directly transfer their contents into the cells by membrane fusion. Therefore, these liposomes can be used as drug carriers for specific purposes.^45,84,85^

###  pH-sensitive liposomes

 To date, various triggered releasemodels are widely researched and reported in order to increase the therapeutic index of pharmaceutical or other materials encapsulated within liposomes. Liposome composition can be modified to obtain triggered release in response to environmental conditions. The pH-sensitive liposomes are designed to control the release of their contents in response to acidic pH of the endosomal system. These liposomes have obviously improved the intracellular delivery of a variety of materials such as anti-cancer drugs, toxins, proteins, and DNA.^86-88^ The typical lipids used to prepare pH-sensitive liposomes are phosphatidylethanolamine (PE) and its derivatives including; diacetylenic phosphatidylethanolamine (DAPE), phosphatidylethanolamine (POPE) and DOPE. They are mixed with the compounds containing an acidic group that acts as a stabilizer at neutral pH. DOPE is usually combined with gently acidic amphiphiles such as oleic acid, CHEMS, and palmitoyl homocysteine.^89,90^ The most commonly used lipid combination is DOPE with CHEMS. Recently, a pH-responsive liposome has been prepared from 3ß-[N-(N’,N’-dimethylaminoethane)-carbamoyl]cholesterol hydrochloride (DC-liposome) for endosomal escape mediated drug delivery. Doxorubicin-loaded DC-liposome has exhibited higher cytotoxicity effect than free drug which supporting the endosomal escape of pH-responsive DC-liposome.^91^

 The pH-sensitive liposomes are stable at neutral pH. In this condition, amphiphilic acid molecules cause the electrostatic repulsion between carboxylate and phosphate groups resulting in the formation of lamellar phases. However, an acidic medium (in pH less than the normal physiological value), either in endosomal vesicles or in the extracellular tumor environment, causes the protonation of the carboxylate groups triggering a transition from lamellar to hexagonal phase leading the release of loaded drugs.^89,92^ The surface of pH-sensitive liposomes can be coated by PEG to prolong the circulation time. Therefore, the liposomes are prevented from rapid clearance via the reticuloendothelial system (RES).^93^

###  Cationic liposomes

 Cationic liposomes are vesicles that are constructed from positively charged lipids and have increasingly been used in gene therapy because of their interactions with negatively charged DNA.^94-97^ It is notable that the negatively charged genetic material is not encapsulated in liposomes but form complex with cationic empty liposomes by electrostatic interactions whereas total surface charge of DNA/liposome remains positive.^81^ DNA-cationic liposome complexes (lipoplexes) enter the cell by fusion with the plasma or endosome membrane. Conventional liposomes are negatively charged and may release their contents in the circulation and/or extracellularly after interaction with blood components and tissues due to their weak affinity for cell membrane. However, unlike these vesicles, cationic liposomes with positive charge are highly interactive with cells (with negatively charged biological membrane) and can deliver contents into cells by fusion with cell membranes. They are usually constructed from a neutral phospholipid (DOPE) and a positive derivative such as stearylamine, dimethyldioctadecylammonium bromide, dimethyl-aminoethane carbamoyl cholesterol (DC-chol), and Dioleoyl-3-trimethylammonium propane (DOTAP).^98,99^

###  Temperature-sensitive liposomes

 The temperature-sensitive liposomes (TSLs) are vesicles that their content release behavior is controlled with temperature changes. TSLs rapidly release the loaded drug at few degrees above physiological temperature or hyperthermic conditions.^100-103^ The release of encapsulated hydrophilic drugs is related to the melting phase transition temperature (Tm) of the lipid bilayer; the temperature that the structure of the lipid bilayer changes from solid gel phase to liquid-crystalline phase. In the liquid-crystalline phase, the membrane of TSLs is more permeable to water and hydrophilic drugs than in the gel phase. In the most TSLs, the major component for liposome formulation is 1,2-dipalmitoyl-sn-glycero-3-phosphocholine (DPPC) with Tm of 41.4°C. To prevent the drug leakage at body temperature, DPPC can be mixed with small amounts of other phospholipids, such as 1, 2-distearoyl-sn-glycero-3-phosphocholine (DSPC; Tm = 54.9°C). The composition of the mixed phospholipids specifies the Tm of the formulation. Further, TSLs can be constructed by modification of conventional liposomes with thermosensitive polymers.^104-106^ TSLs in combination with local hyperthermia or high intensity focused ultrasound are concerned as effective route for external targeting of anti-cancer drugs to solid tumors.^107,108^

###  Stealth liposomes 

 Stealth liposomes (long circulating liposomes) namely “second-generation liposomes” are obtained by modifying the surface of the vesicles with an inert molecule.^29^ At first, liposomes with modified surfaces were developed using several molecules, such as glycolipids or sialic acids. However, with inclusion of the synthetic polymer polyethylene glycol (PEG) in the liposome composition, the long-circulating pegylated liposomes as a new generation were emerged. It has been proved that such surface modification extends blood-circulation time of liposomes and stabilizes these nanocarriers by minimizing their interaction with the RES.^45,109^ So far, this technology has been used to formulate a large number of liposomes containing various drugs or other biomolecules with high efficiency and activity. Moreover, by combining of the terminal PEG with appropriate compound, long-circulating liposomes can be synthesized to target on specified cells.^109^

###  Magnetoliposomes

**Figure 2:**
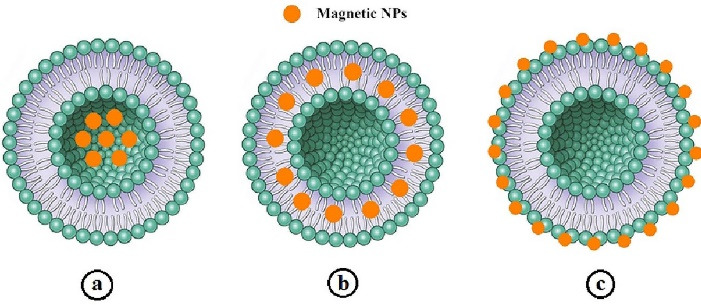
Schematic illustration of three approaches in associating of SPIONs to the liposomes: a) Encapsulation directly within liposome lumen, b) embedding in between the lipid bilayer, and (c) directly conjugating to the liposome surface^115^

 Combination of liposomes and SPIONs or other magnetic nanoparticles,^110,111^ that called magnetoliposomes (MLs) is an interesting strategy creating vesicles with the potential for the application in controlled drug delivery systems and diagnostic imaging. They are promising nanocarriers for the development of the selective and site-specific drug delivery systems in the cancer therapy which can effectively deliver the drug towards tumor cells by applying a magnetic field.^112,113^ MLs are widely exploited as contrast agents in magnetic resonance imaging and as chemotherapeutic agents.^114^ There are three different approaches in associating the SPIONs to liposomes: (i) encapsulation of magnetic nanoparticles directly within the liposome lumen,^115-117^ (ii) embedding them in between the lipid bilayer,^118-120^ and (iii) directly conjugating magnetic nanoparticles to the liposome surface.^121^ The different types of MLs are depicted in Figure 2.

###  Photosensitive liposomes

 In the contrary to internal triggerable liposomes like pH-sensitive liposomes, external triggering system exploit the outside factors such as light, heat or magnetic field to release the liposomal cargo. On the other hand, the thermo-sensitive liposomes undergo phase transition of the phospholipids, while photo-triggerable liposomes are composed of a light-sensitive group engineered into the vesicle. Function of light-triggering liposome is based on two approaches: (i) photo-destabilization of liposome membrane to promote cargo release and (ii) light absorption of metal nanoparticles such as gold nanoparticles.^122^ Different photosensitive molecules can induce membrane destabilization and permeabilization. They can locate in the liposomal structure according to their intrinsic polarity. Phospholipid molecule modification can be performed in potential sites, namely, head group, glycerol backbone and fatty acyl chains.^123-126^ The various mechanisms for cargo release from photosensitive liposomes including; light-induced oxidation, photocrosslinking, photoisomerization, photocleavage, and photothermal release have been extensively reviewed by Miranda and Lovell.^124^

**Figure 3:**
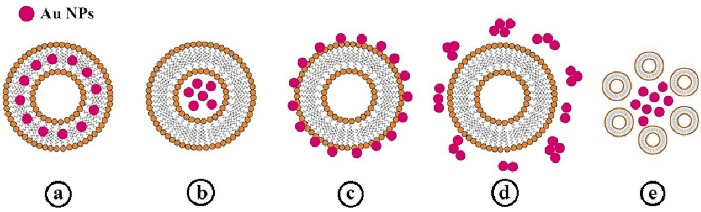
Different types of GNPs incorporation in liposome structure: (a) in the lipid bilayer, (b) in the lumen, (c) on the surface of liposomes, (d) free in the liposome solution, and (e) aggregate with liposomes^16^

 In case of incorporation of metal nanoparticles like GNPs in liposomal structure, they can localize within lipid bilayer, into the lumen, and on the surface of liposomes, aggregate with liposomes or be free in liposome solution (Figure 3). With irradiation of liposomes, GNPs convert the photo energy to thermal energy, inducing the instability of liposome membrane; therefore, the entrapped drug is released. Photo-responsive liposomes are powerful carriers for topical and transdermal drug delivery to superficial tissues like skin, eyes, and mucous membranes.^16,127^

## Liposomes as targeting nanocarriers

 Cancer therapy using liposomes can be accomplished through two main approaches including; passive and active targeting.

###  Passive targeting of liposomes

**Figure 4:**
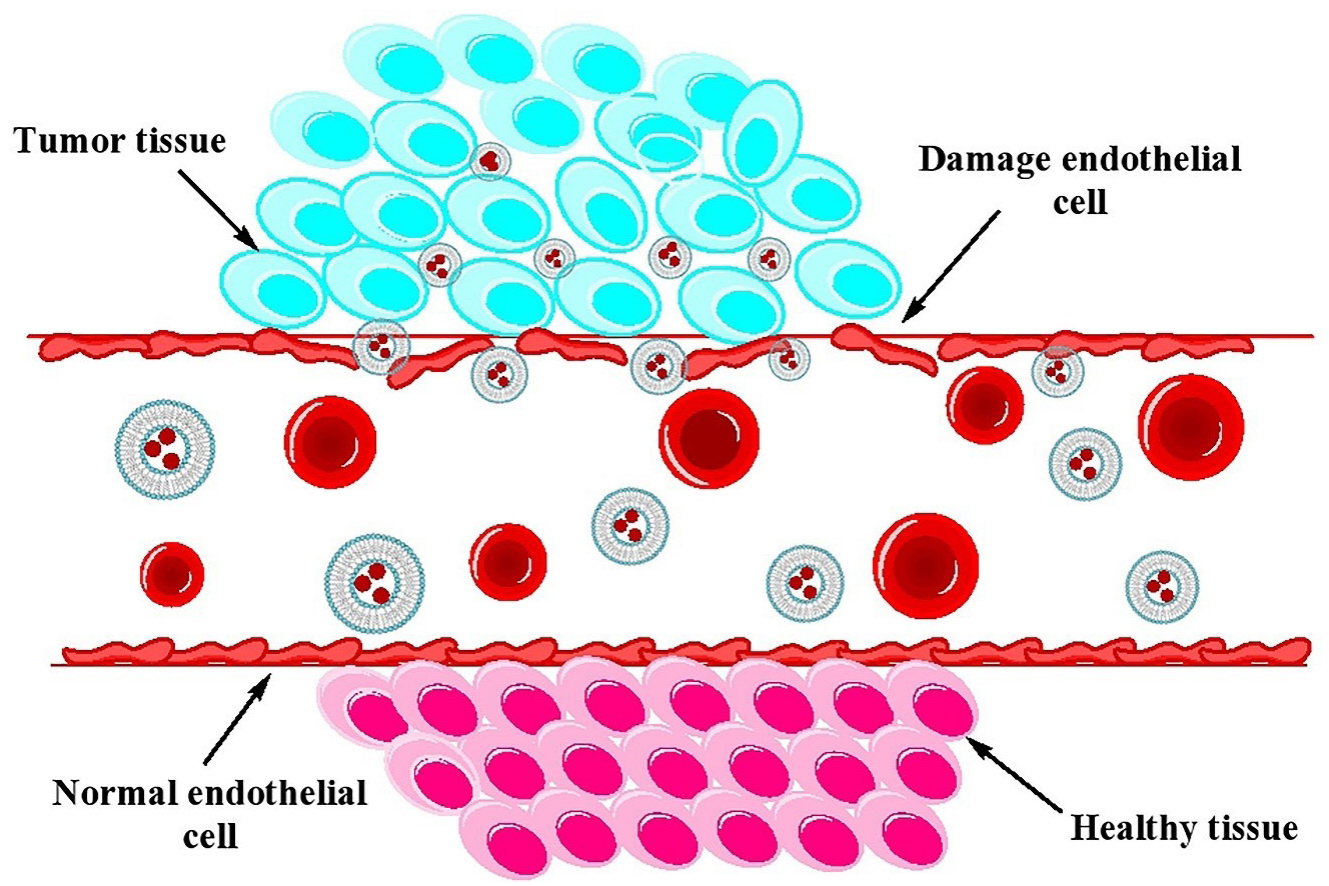
Schematic illustration of the mechanism of passive targeting

 In passive targeting, the nanocarriers are transported into the tumor interstitium and cells through leaky tumor capillary fenestrations by convection or passive diffusion.^128^ In general, all nanoparticle-based drug delivery systems use the tumor characteristics for targeting. The angiogenesis phenomenon in tumor tissues causes the irregularity of endothelial cells with pore sizes of 100 nm to 2 μm. The different pore sizes between the endothelial cells of the tumor microvasculature and the tighter structures of normal cells causes the nanoparticles such as liposomes have better and more access to the cancerous sites. The “enhanced permeability and retention effect” (EPR) causes the increased drug delivery to the affected tissues with a much less return of the fluids to the lymphatic circulation.^129,130^ All nanocarriers benefit from the EPR effect in passive targeting so that of drug-loaded nanocarriers in tumor site are 10-50 folds higher than in normal tissue within 1-2 days.^131^ The nanocarriers must have at least three characteristics to exploit in passive drug delivery system: (i) The size of nanocarrier should be much less than 400 nm, and being in the range of 10-100 nm which is ideal for efficient extravasation to tumor site, (ii) having neutral or anionic charge for the nanocarriers is necessary to avoid the renal elimination, and (iii) the nanocarriers should be protected from the RES.^128,131,132^ The mechanism of passive targeting is illustrated in Figure 4. A number of successful results have been obtained from passive targeting property of liposomes in cancer therapy.^133-138^

###  Active targeting of liposomes

**Figure 5:**
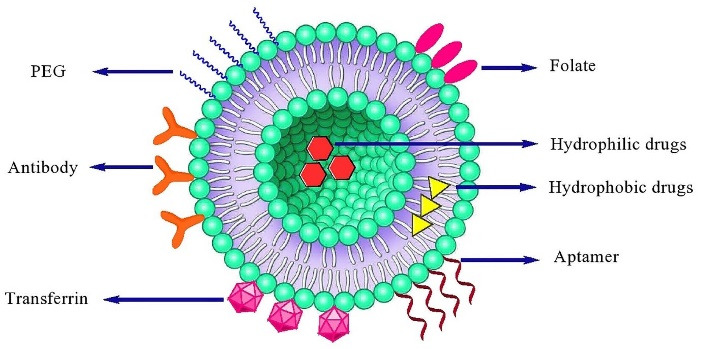
A schematic presentation of various ligands used in liposome targeting

**Figure 6:**
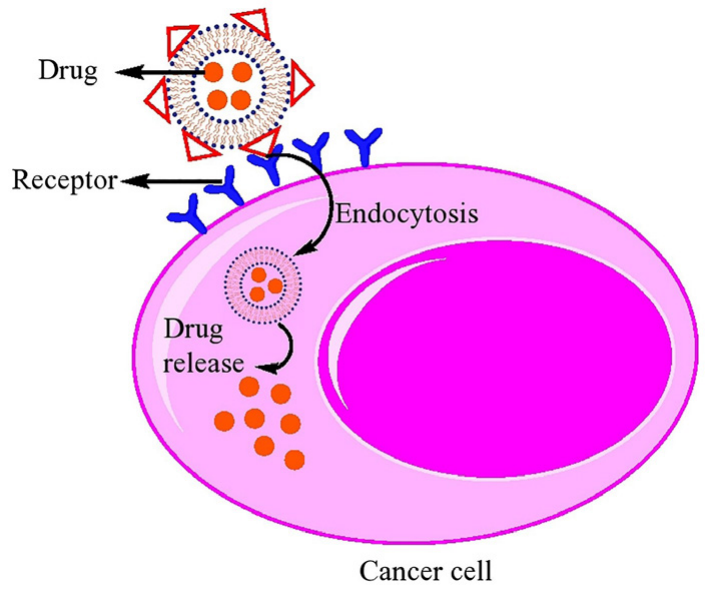
Schematic representation of active targeting using surface engineered liposomes

 The reduction of drug toxicity and increase the therapeutic index can be implemented by the site-specific delivery. Nanocarriers can reach tumor microenvironment passively through the EPR effect, whereas the surface engineered nanomedicine acts through binding to the receptors over-expressed by cancer or angiogenic endothelial cells such as epidermal growth factor, fibroblast growth factor, folate, transferrin, and nuleolin receptors (Figure 5). Targeting these overexpressed receptors to increase the anti-cancer agents up taken by cancer cell as well as accumulation in cancer microenvironment is a vital approach.^31^ Surface modification of a variety of nanocarriers such as liposomes with antibodies specified for cancer cells, is a more common method. In addition to antibodies, the other molecules or biomaterials with the various strategies for the conjugation have been attached to PEGylated liposomes which enabling them to be actively taken up by the target cells *via *receptor-mediated endocytosis.^128,139,140^ Active targeting and efficient ligand receptor interaction are related to some factors such as the extent of expression of the receptor on tumor cells relative to non-target cells, availability of the receptor on the surface of target cells, the internalization rate, and heterogeneous expression of tumor receptor.^141^ Monoclonal antibodies, transferrin,^1^ folic acid,^34^ and aptamers^142,143^ have been frequently used for surface modification of nanocarriers such as liposomes.^128,130,144,145^ A schematic presentation of entrance of these ligands into the cell is shown in Figure 6. A number of researches have been performed in this area. Recently, functionalization of liposomal surface and targeting strategies in treatment of solid tumors are extensively investigated. Recently, mannosylated liposomes have been developed to encapsulate Chlorogenic acid as a targeted delivery system to tumor-associated macrophages (TAMs) for cancer immunotherapy. It has been reported that chlorogenic acid-loaded liposomes conjugated with mannose exhibited superior accumulation in tumors through the mannose receptor-mediated TAMs-targeting effects.^146^ Cancer cells overexpress α5β1, αvβ3 and αvβ5 integrins and it has been observed that the cyclic RGD (cRGD) can strongly attach to αvβ3 and αvβ5 integrins on cancer cells. cRGD-PEG liposomes loaded miR-34a have been developed for suppressing microRNA in breast cancer cells.^147^ In one study, it has been reported that tyrosine-modified irinotecan-loaded liposomes exhibited more cellular uptake in MCF-7 and BxPC-3 cells due to highly expressed ATB^0,+^ and LAT1 in cancer cells.^148^ In another study, the liposomes modified by glutamic hexapeptide and folic acid were designed for bone metastatic breast cancer. The results showed that paclitaxel loaded in co-modified liposomes presented high stability, more hydroxyapatite binding efficiency and also improved cytotoxic activity of the drug.^149^ Some of the more recent published works in this field are summarized in Table 2.

**Table 2:** Some recently published researches in active targeting of liposomes for cancer therapy

**Targeting ligand**	**Drug**	**Liposome type**	**Preparation method**	**Loading method**	**Cancer treated**	**Ref.**
Monoclonal antibodies	Glycosylated paclitaxel	Immunoliposomes	Thin Film	Passive	Ovarian	^150^
Monoclonal antibodies	Curcumin	Cationic liposome	Thin Film	Passive	Breast	^151^
Monoclonal antibodies	Doxorubicin	PEG-liposome	Ethanol injection	Passive	Breast	^152^
Monoclonal antibodies	Doxorubicin	PEG-liposome	Thin Film	Passive	Breast	^153^
Folate	Oleuropein	PEG-liposome	Thin Film	Passive	Prostate	^154^
Folate	Gold nanorods and doxorubicin	PEG-liposome	Thin Film	Passive	Breast	^155^
Folate	Rapamycin	PEG-liposome	Thin Film	Passive	Bladder	^156^
Folate	Arsenic trioxide	PEG-liposome	Thin Film	Active	Cervical	^157^
Transferrin	Doxorubicin and Sorafenib tosylate	PEG-liposome	Thin Film	Passive	Breast	^158^
Transferrin	Doxorubicin	Cationic liposome	Ethanol injection	Passive	Glioma	^159^
Transferrin	Plumbagin	PEG-liposome	Thin Film	Passive	Carcinoma, glioblastoma	^160^
Transferrin	Resveratrol	PEG-liposome	Thin Film	Passive	Glioblastoma	^161^
Aptamer	A-particle generator 225Ac	PEG-liposome	Thin Film	Passive	Prostate	^162^
Aptamer	All-trans retinoic acid	PEG-liposome	Thin Film	Passive	Bone	^163^
Aptamer	MiR-139-5p	Cationic Liposome	Thin Film	Passive	Colorectal	^164^
Aptamer	Paclitaxel and siRNA	Cationic Liposome	Thin Film	Passive	Breast	^165^
Mannose	chlorogenic acid	PEG-liposome	Thin Film	-	glioblastoma	^146^
RGD	microRNA	PEG liposomes	Thin Film	-	Breast	^147^
Amino acid	irinotecan	Liposome	Thin Film	-	Breast	^148^
Glutamic hexapeptide and folic acid	paclitaxel	liposome	Thin Film	-	Bone metastatic breast cancer	^149^

## Methods for encapsulating materials into liposomes

 Various methods of liposome loading largely depend on the physicochemical characteristics of the loaded agents. In general, the encapsulation strategies are divided in two categories: passive and active loading.^166,167^

###  Passive loading

 In the all techniques where the lipids and encapsulating agents are introduced in an aqueous buffer solution and the entrapment is achieved while the liposomes are being formed, a passive trapping is occurred. Passive loading of pharmaceutical agents into the liposomes are implemented by two ways including (i) entrapment in liposomal membrane (bilayer) by hydrophobic interaction, electrostatic interaction or combination of these two mechanisms, or (ii) entrapment of hydrophilic substances such as salts (ionic compounds), amino acids, antibiotics, and proteins in intra-liposome aqueous phase (shown in Figure 1). However, the lipophilic substances are added in the organic solvent containing lipids, before formation of liposomes.^168^ In both routes, encapsulation is occurred simultaneously with the lipid self-assembly and liposome formation.^169^ In this method the loading efficiency, and therefore, drug/lipid ratio (D/L) is usually low (10-50%). However, some additional procedures such as freeze-thaw and dehydration-rehydration can provide higher encapsulation efficiencies. Besides, encapsulation efficiency depends on some factors such as the lipid amount and concentration, and the solubility of the entrapping agent in aqueous phase.^166,170-172^

###  Active loading

 Passive encapsulation method presents some disadvantages including; low entrapment efficiency (20-30%), non-loaded drug loss, organic solvent impurities, and fast release of drug. Hydrophilic small molecules are usually passively loaded during the phospholipid self-assembly. However, amphiphilic substances can be actively loaded into liposomes after liposome formation without diffusing back out. In active encapsulation, the molecules cross through the lipid bilayer into the internal aqueous compartment of liposomes, and cannot diffuse back out or return into the external aqueous solution. The active loading methods are typically based on two phenomena: (i) a given lipophilic molecule diffuse the lipid bilayer and gain a charge upon entering the liposomal core and (ii) the molecule as an ion cannot be able to cross the bilayer and accumulation of the encapsulated agent is achieved.^167,173^ Some frequently used active drug loading methods are summarized in the following sections.

####  The pH gradient method

 In this method, penetration of the drug into the preformed liposomes is driven by a transmembrane pH gradient. In order to achieve the efficient active loading, the aqueous solubility of the encapsulating drug and the presence of ionizable functional groups in its structure (e.g. amine group in weak bases) are necessary.^174^ In the pH of the extra-liposome aqueous phase the drug exists in the unionized form and is able to migrate across the liposome bilayer. Upon translocation into the liposomal core, the drug changes to ionized form due to the differing pH and retained there. Thus, for amphiphilic drugs which are weak bases or acids, a pH gradient can be the driving force to translocate and retain in liposomes. A pH gradient of 3 units can cause a 1000-fold higher concentration of a substance within the liposome core in comparison to the external aqueous phase.^167,173-175^

####  Citrate method

 In this approach pH of intra-liposomal core is 4 owing to the presence of citrate buffer and extra-liposome aqueous phase has a pH equal to 7.4 which is adjusted with HEPES buffer.^176^ Therefore, a proton gradient is observed when substances such as biogenic amines and anti-cancer doxorubicin are present in extra-liposomal aqueous solution. In the presence of HEPES buffer (pH 7.4), the compounds containing the amine groups are in the neutral form and therefore, are able to cross the lipid bilayer. By entering the amines inside the liposome, they produce low-soluble citrate salts due to the presence of citrate ions. This method has been used for remote loading of anthracycline into the liposomes with coffee bean liposome appearance.^177,178^ Citrate method was successfully exploited in commercially manufacturing of doxorubicin and daunorubicin products namely Myocet and DaunoXome, respectively.^173^

####  Ammonium sulfate gradient method

 The remote loading strategy that called transmembrane ammonium sulfate gradient method was introduced by Haran et al. for the encapsulation of amines.^179^ In this method, there is no need to prepare the liposomes in the acidic pH and alkalinize of extra-liposomal aqueous phase.^180^ Ammonium ion gradient is generated *via *its counter-ion sulfate which stabilizes anthracyclines by aggregation and gelation as anthracycline sulfate salt. Firstly, the empty liposomes are formed in ammonium sulfate solution using thin-layer method. Then, the liposomes are dialyzed in PBS solution to form ammonium sulfate gradient inside and outside of liposomes. After the formation of ammonium sulfate gradient, remote encapsulation is carried out by incubation of liposomes with drug solution. The neutral ammonia molecules (permeability coefficient 0.13 cm/s) are diffused towards extra-liposomal solution and left behind a proton due to the higher concentration of ammonium ions in aqueous core of liposome; therefore, the pH gradient is formed. The drug including; amine group in its neutral form (in pH 7.4 buffer) penetrates the bilayer and precipitation of the drug as sulfate salt is occurred. Doxil as the first commercially available long circulating liposomal doxorubicin was produced using ammonium sulfate gradient method.^173,179-181^

####  Calcium acetate method

 The transmembrane calcium acetate method is based on different permeability coefficients of acetic acid and calcium ions. In this technique, the blank liposomes are prepared in calcium acetate solution. Whereas the calcium ions remain in the liposomal aqueous core, the acetic acid molecules act as proton shuttles. This generates a pH gradient, with higher pH value inside the liposomes, results in entrapping the weak amphiphilic acid molecules inside the liposomes in a way similar to that of weak amphiphilic bases.^173,181,182^ In the another method, similar to the aforementioned strategy, arsenic trioxide as an anti-cancer agent has been actively loaded into the liposomes that contain acetate salts of bivalent cations such as Co(II), Ni(II), Cu(II), and Zn(II). The external neutral As (OH)_3_ penetrates across the bilayer and forms the low-soluble heavy metal- arsenite complex in the liposomal core. On the other hand, the released protons associate with the acetate anions produce the weak acid (HOAC) which diffuses out of the liposome. Both phenomena: the formation of insoluble nickel (II) arsenite compound and the diffusion of the acetic acid out of the liposome drive drug uptake.^183^

####  Ionophore-mediated method

 This protocol involved the use of ionophore agents such as antibiotic nigericin or A23187 which mediate the exchange of K ^+^ and H ^+^ across the liposomal bilayer generating pH gradient of about 2 units. When, loaded liposomes with K_2_SO_4_ are placed in the K ^+^ -free aqueous phase containing an ionophore, the pH of the intra-liposomal aqueous solution decreases due to the release of potassium and the entry of protons.^184^ This pH decrease results in the active encapsulation of weak bases. It is notable that nigericin cause a one-for-one exchange of K ^+^ for H ^+^, whereas A23187 makes it possible to move two protons per every divalent metal cation such as Ca^2 +^, Mn^2 +^, or Mg^2 +^. In the use of divalent cations, the presence of EDTA as external chelator is required to bind with the released cations and complete the uptake process as well as prevent aggregation of liposomes.^173,185^

####  EDTA gradient method

 It has been reported that EDTA can form low soluble precipitates with anthracyclines or other weak bases and the formation of low soluble EDTA-drug complexes inside of liposomes can lead to increased drug encapsulation and retention. The protocol is especially suggested for the encapsulation of idarubicin because of its very low solubility in EDTA solutions at acidic pHs. In this case, the liposomes are formed *via* hydration of the lipid film with EDTA disodium salt or EDTA di-ammonium salt solution and then, idarubicin hydrochloride solution is added to the liposomal suspension. The accumulation of idarubicin in liposomes is pH-dependent, so that in higher external pH (8.5) and lower internal pH (4) the drug is better accumulated and higher encapsulation efficiency is achieved.^173,186^

####  Phosphate gradient method

 The main concept in transmembrane phosphate gradient strategy is the same as in the case of other pH gradient methods. In this case, the liposomes are prepared in (NH_4_)_2_HPO_4_ solution. It is reported that a near 100% doxorubicin accumulation inside the liposomes *via* both protonation and precipitation of drug have been observed as in the case of other gradients. Besides, in pHs close to physiological level no drug leakage is observed from the liposomes. However, in acidic extra-liposomal medium accelerated drug leakage is achieved. It was suggested that doxorubicin can be retained in the hydrophilic liposomal core by protonation and precipitation, or incorporated in the bilayer. This remote loading process depends on various factors such as intra-liposomal salt concentration and pH value.^173,187^

####  Solvent-assisted active loading method

 In all the aforementioned remote loading methods the compounds with high solubility and membrane permeability are solubilized in the exterior aqueous phase and penetrate through the liposome bilayer into the internal aqueous phase. However, a large number of drugs have low-water solubility and are passively encapsulated in the lipid bilayer of liposomes. The solvent-assisted active loading technology (SALT) has been used for remote encapsulating of poorly soluble drugs in the liposomal core in order to achieve the better loading efficiency and formulation stability. In this technique, a small volume (~5 v/v %) of a water miscible solvent is added to the loading solution for complete dissolution of the compound. Then, the dissolved compound diffuses into the internal compartment of liposome and interacts with a precipitating agent to form low-soluble precipitate. The solvent can be eliminated using dialysis or gel filtration techniques. It is reported that a rapid and complete encapsulation with high D/L ratio and improved circulation half-life are achieved by exploiting this method.^188^

## Medical applications of liposomes

 Liposomes can be used in the various clinical fields including; therapeutic systems, medical imaging, and cosmetic products which have been summarized below^13^:

###  Therapeutic systems

 Liposomes are used in many fields of medical treatment such as cancer therapy, anti-infective therapy, protein or peptide drug delivery, gene delivery, macrophage activation, and vaccination.^13,15,27,45^ The first application of liposomes as drug delivery system was the delivery of chemotherapeutic small molecules.^15^ A large number of drugs are formulated into the liposomes taking advantage of high therapeutic efficiency and low systemic toxicity compared with the free drug. Anti-infective drugs and anti-tumor drugs are two major classes of small molecules that can be loaded in liposomal formulations. After approval of liposomal systems for delivery of small molecules, delivery of macromolecules such as nucleic acid-based therapeutics (gene therapy agents) was noticed. Nucleic acid-based materials with high-molecular weight and highly charged molecules cannot cross cell membranes by passive diffusion. Moreover, applications of these materials as therapeutic agents are limited by their rapid enzymatic degradation, low selectivity, and poor cellular uptake. Lipoplex is a complex between negatively charged nucleic acid-based material and cationic liposome which can enter the cell *via* fusion with the plasma membrane. Thereby, gene therapy is performed through these liposomal formulations. Protein or peptide based therapeutic agents including; enzymes, peptide hormones, and cytokines are the other class of drugs that can be encapsulated in liposomes.^189,190^ Incorporation of these agents into the liposomes resulted in some advantages such as improving therapeutic activity of protein and peptide drugs, reducing their side effects and modulating the immune response towards these proteins and peptides.^45^

###  Diagnostic imaging

 Apart from the method used, in diagnostic imaging the appropriate intensity of signal from an area of interest is needed to differentiate specified structures from surrounding tissues. On the other hand, molecular imaging has an important role in diagnosis and treatment tracing of diseases. Liposomes can be targeted to specified disease tissues by combining with specific targeting ligands and imaging molecular probes. These probes are loaded with liposomes in four ways: (i) incorporating into the liposome during its formation, (ii) penetration into the lipid bilayer of preformed liposome, (iii) encapsulation into the preformed liposome *via* various active methods, and (iv) attaching on the surface of preformed liposome.^45,191,192^

###  Cosmetics

 Liposomes have been considered in the delivery of ingredients in cosmetics due to their unique physicochemical properties. Incorporation of liposomes in cosmetic formulations has shown some advantages such as increasing in skin moisture, improving the cell membrane fluidity, and causing deep penetration of oil or water-soluble cosmetic ingredients through the skin. Liposomal cosmetics have been manufactured by famous company namely Christian Dior, for first time in 1987. After that, some other liposomal cosmetic formulations are reported and manufactured.^13,45^

## Future prospective of liposomal formulations

 Today, many approved liposomal and nano-liposomal products entered the commercial market. Some of these products are presented in Table 3.^193-196^ However, there are huge challenges in their clinical translation. It seems that the most important field of research ahead is related to solving problems related to the targeted liposomal formulations. Many of these nanocarriers have shown more efficiency in animal and *in vitro* studies; however; a few of them have been entered to clinical trials, and there is not any investigationand evaluation guideline for active targeting liposomal formulations in cancer treatment.^20^ To achieve the successful clinical use of these formulations, many problems have to be solved. It has been shown that a number of factors such as liposome size and charge, type and amount of ligand, the ligand binding with the serum proteins, and the elimination by the body immune system can influence on the function of targeted liposomes.^197-202^ Owing to the excellent clinical potential of targeted liposomes to improve the therapeutic index of drugs, further research is required for their clinical applications.

**Table 3:** List of some approved liposomal drugs

**Trade name**	**Chemical name**	**Clinical uses**	**Ref.**
Ambisome	Amphotericin B	Sever fungal infections	^26^
DaunoXome	Daunorubicin	Kaposi's sarcoma	^26^
Myocet	Doxorubicin	Breast cancer	^13^
DepoDur	Morphine	Pain following surgery	^13^
DepoCyt	Cytarabine	Neoplastic meningitis, lymphomatous meningitis	^13^
Visudyne	Vertporfin	Macular degeneration, pathologic myopia, ocular histoplasmosis	^170^
Abelcet	Amphotericin B	Sever fungal infections	^26^
Amphotec	Amphotericin B	Sever fungal infections	^26^
Doxil	Doxorubicin	Kaposi's sarcoma, ovarian and breast cancer	^24^
Onivyde	Irinotecan	Pancreatic cancer	^13^
ELA-Max	Lidocaine	Skin diseases	^144^
Newcastle	Disease vaccine	Chicken	^144^
Epaxal	A vaccine	Hepatitis	^144^
Lipodox	Doxorubicin	Anti-cancer	^170^
Marqibo	Vincristine	Acute lymphoblastic leukemia	^13^
EvacetTM	Doxorubicin	Metastatic breast cancer	^203^
Avian retrovirus vaccine	Killed avian retrovirus	Chicken pox	^203^
Novasome®	Smallpox vaccine	Smallpox	^203^
Topex-Br	Terbutaline sulphate	Asthma	^203^
Alectm	Dry protein free powder of DPPC-PG	Expanding lung diseases in babies	^203^
Ventustm	Prostaglandin-E1	Systemic inflammatory diseases	^203^
Fungizone®	Amphotericin-B	Fungal infections, Leishmaniasis	^203^
VincaXome	Vincristine	Solid Tumors	^204^
Autragen	Tretinoin	Kaposi’s sarcoma	^204^
Shigella Flexneri 2A vaccine	Shigella flexneri 2A	Shigella flexneri 2A infections	^204^
Nyotran	Nystatin	Systemic fungal infections	^204^

## Conclusion

 Liposomes have been recognized as therapeutic carriers in very diverse clinical fields because of their unique physicochemical properties. They are the first nano-delivery systems that some of them are already successfully translated into the clinical use, and some liposomal formulations are approved or under clinical trials. Employing of liposomes as drug delivery systems provide a platform for delivering of drugs with reducing side effects and increasing their efficacy, solubility, and bioavailability. Despite the improvements made to these carriers to reduce their adverse effects and increase the therapeutic index of the cargo, investigations to fabricate the liposomes with fewer deficiencies are ongoing. A number of synthesis methods have been developed to obtain liposomes with various structure, size, and polydispersity. Ethanol injection technique is one of the interesting methods for scaling-up production of liposomes due to simplicity, fast implementation, and reproducibility. To overcome some practical challenges such as precise process control, poor reproducibility, and inefficient use of materials and reagents novel strategies such as microfluidic and SCF based methods have been designed for preparation of liposomes. Microfluidic iseffective reproducible method for scale-up of liposomes owing to achieve more control over the physical properties including; size distribution, lamellarity, and high encapsulation efficiency. Moreover, solvent free and pharmaceutical grade liposomes having a narrow particle size distribution can be produced by SCF method. Fabrication of the PEGylated liposomes is the important modification to solve the problem of uptake by RES and rapid clearance from bloodstream. However, the selective delivery of these liposomes to the action site is limited. Today, research in fabrication of stimuli-sensitive and functionalized liposomes are two forefront fields to increase of their target specificity. Besides, to overcome to the passive loading drawbacks such as low entrapment efficiency, non-loaded drug loss, and fast release of drug, several active loading methods have been developed. Furthermore, in order to enhance internalization of liposomes by the specified tissues, their surfaces can be modified with targeting ligands such as transferrin, integrins, polysaccharides, folic acid, aptamers and antibodies. However surely, liposomes have found their place in the modern pharmaceutics and their use is increasing day by day. Nowadays, many liposomal anti-cancer drugs have been used in the treatment of breast, ovarian cancers, and sarcoma. Due to the potential clinical applications of liposomes, challenges such as therapeutic and loading efficiency, stability, and scale up of industrial production with more clinical success, needs further investigation.

## Acknowledgments

 This work was supported by Ahvaz Jundishapur University of Medical Sciences, Ahvaz, Iran.

## Author Contributions

**Conceptualization:** Hanieh Abbasi, Maryam Kouchak, Zohreh Mirveis, Fatemeh Hajipour, Mohsen Khodarahmi.

**Formal Analysis:** Nadereh Rahbar, Hanieh Abbasi.

**Investigation:** Hanieh AbbasiD, Maryam Kouchak, Zohreh Mirveis, Fatemeh Hajipour, Mohsen Khodarahmi.

**Methodology: **Nadereh Rahbar, Hanieh Abbasi.

**Project administration: **Nadereh Rahbar, Somayeh Handali.

**Software: ** Nadereh Rahbar, Hanieh Abbasi.

**Supervision:** Nadereh Rahbar, Somayeh Handali.

**Validation: ** Nadereh Rahbar, Hanieh Abbasi.

**Visualization:** Nadereh Rahbar, Somayeh Handali.

**Writing – original draft: **Hanieh Abbasi. Nadereh Rahbar.

**Writing – review & editing: **Nadereh Rahbar, Somayeh Handali.

## Ethical Issues

 Not applicable.

## Conflict of Interest

 The authors declare that they have no competing interests.

## Supplementary Files

Supplementary file 1 contains Figures S1-S5.

## References

[1] Moghimipour E, Rezaei M, Kouchak M, Ramezani Z, Amini M, Ahmadi Angali K (2018). A mechanistic study of the effect of transferrin conjugation on cytotoxicity of targeted liposomes. J Microencapsul.

[2] Singh R, Lillard JW Jr (2009). Nanoparticle-based targeted drug delivery. Exp Mol Pathol.

[3] Wu PT, Lin CL, Lin CW, Chang NC, Tsai WB, Yu J (2018). Methylene-blue-encapsulated liposomes as photodynamic therapy nano agents for breast cancer cells. Nanomaterials (Basel).

[4] Letchford K, Burt H (2007). A review of the formation and classification of amphiphilic block copolymer nanoparticulate structures: micelles, nanospheres, nanocapsules and polymersomes. Eur J Pharm Biopharm.

[5] Lee CC, MacKay JA, Fréchet JM, Szoka FC (2005). Designing dendrimers for biological applications. Nat Biotechnol.

[6] Bosman AW, Janssen HM, Meijer EW (1999). About dendrimers: structure, physical properties, and applications. Chem Rev.

[7] Mahmoudi M, Sant S, Wang B, Laurent S, Sen T (2011). Superparamagnetic iron oxide nanoparticles (SPIONs): development, surface modification and applications in chemotherapy. Adv Drug Deliv Rev.

[8] Tang F, Li L, Chen D (2012). Mesoporous silica nanoparticles: synthesis, biocompatibility and drug delivery. Adv Mater.

[9] Ghosh P, Han G, De M, Kim CK, Rotello VM (2008). Gold nanoparticles in delivery applications. Adv Drug Deliv Rev.

[10] Juzenas P, Chen W, Sun YP, Coelho MA, Generalov R, Generalova N (2008). Quantum dots and nanoparticles for photodynamic and radiation therapies of cancer. Adv Drug Deliv Rev.

[11] Belin T, Epron F (2005). Characterization methods of carbon nanotubes: a review. Mater Sci Eng B.

[12] Hossen S, Hossain MK, Basher MK, Mia MNH, Rahman MT, Uddin MJ (2019). Smart nanocarrier-based drug delivery systems for cancer therapy and toxicity studies: a review. J Adv Res.

[13] Li M, Du C, Guo N, Teng Y, Meng X, Sun H (2019). Composition design and medical application of liposomes. Eur J Med Chem.

[14] Sousa I, Rodrigues F, Prazeres H, Lima RT, Soares P (2018). Liposomal therapies in oncology: does one size fit all?. Cancer Chemother Pharmacol.

[15] Abu Lila AS, Ishida T (2017). Liposomal delivery systems: design optimization and current applications. Biol Pharm Bull.

[16] Mathiyazhakan M, Wiraja C, Xu C (2018). A concise review of gold nanoparticles-based photo-responsive liposomes for controlled drug delivery. Nanomicro Lett.

[17] Anwekar H, Patel S, Singhai AK (2011). Liposome-as drug carriers. Int J Pharm Life Sci.

[18] Verma P, Ram A, Jha AK, Mishra A, Thakur A (2010). Phosphatidylcholine: a revolution in drug delivery technology. Int J Pharm Sci Res.

[19] Patil YP, Jadhav S (2014). Novel methods for liposome preparation. Chem Phys Lipids.

[20] Yan W, Leung SS, To KK (2020). Updates on the use of liposomes for active tumor targeting in cancer therapy. Nanomedicine (Lond).

[21] Fenske DB, Cullis PR (2008). Liposomal nanomedicines. Expert Opin Drug Deliv.

[22] Xu X, Ho W, Zhang X, Bertrand N, Farokhzad O (2015). Cancer nanomedicine: from targeted delivery to combination therapy. Trends Mol Med.

[23] He H, Lu Y, Qi J, Zhu Q, Chen Z, Wu W (2019). Adapting liposomes for oral drug delivery. Acta Pharm Sin B.

[24] Sercombe L, Veerati T, Moheimani F, Wu SY, Sood AK, Hua S (2015). Advances and challenges of liposome assisted drug delivery. Front Pharmacol.

[25] Belfiore L, Saunders DN, Ranson M, Thurecht KJ, Storm G, Vine KL (2018). Towards clinical translation of ligand-functionalized liposomes in targeted cancer therapy: challenges and opportunities. J Control Release.

[26] Dua JS, Rana AC, Bhandari AK (2012). Liposome: methods of preparation and applications. Int J Pharm Stud Res.

[27] Daraee H, Etemadi A, Kouhi M, Alimirzalu S, Akbarzadeh A (2016). Application of liposomes in medicine and drug delivery. Artif Cells NanomedBiotechnol.

[28] Akbarzadeh A, Rezaei-Sadabady R, Davaran S, Joo SW, Zarghami N, Hanifehpour Y (2013). Liposome: classification, preparation, and applications. Nanoscale Res Lett.

[29] Immordino ML, Dosio F, Cattel L (2006). Stealth liposomes: review of the basic science, rationale, and clinical applications, existing and potential. Int J Nanomedicine.

[30] Arias JL (2013). Liposomes in drug delivery: a patent review (2007-present). Expert OpinTher Pat.

[31] Riaz MK, Riaz MA, Zhang X, Lin C, Wong KH, Chen X (2018). Surface functionalization and targeting strategies of liposomes in solid tumor therapy: a review. Int J Mol Sci.

[32] Kirby C, Clarke J, Gregoriadis G (1980). Effect of the cholesterol content of small unilamellar liposomes on their stability in vivo and in vitro. Biochem J.

[33] Handali S, Moghimipour E, Kouchak M, Ramezani Z, Amini M, Angali KA (2019). New folate receptor targeted nano liposomes for delivery of 5-fluorouracil to cancer cells: strong implication for enhanced potency and safety. Life Sci.

[34] Moghimipour E, Rezaei M, Ramezani Z, Kouchak M, Amini M, Angali KA (2018). Folic acid-modified liposomal drug delivery strategy for tumor targeting of 5-fluorouracil. Eur J Pharm Sci.

[35] Handali S, Moghimipour E, Rezaei M, Ramezani Z, Kouchak M, Amini M (2018). A novel 5-Fluorouracil targeted delivery to colon cancer using folic acid conjugated liposomes. Biomed Pharmacother.

[36] Sułkowski WW, Pentak D, Nowak K, Sułkowska A (2005). The influence of temperature, cholesterol content and pH on liposome stability. J Mol Struct.

[37] Ichihara K, Iwasaki H, Ueda K, Takizawa R, Naito H, Tomosugi M (2005). Synthesis of phosphatidylcholine: an improved method without using the cadmium chloride complex of sn-glycero-3-phosphocholine. Chem Phys Lipids.

[38] Bouchet AM, Frías MA, Lairion F, Martini F, Almaleck H, Gordillo G (2009). Structural and dynamical surface properties of phosphatidylethanolamine containing membranes. BiochimBiophys Acta.

[39] Allen C, Dos Santos N, Gallagher R, Chiu GN, Shu Y, Li WM (2002). Controlling the physical behavior and biological performance of liposome formulations through use of surface grafted poly(ethylene glycol). Biosci Rep.

[40] Garigapati VR, Roberts MF (1993). Synthesis of short chain phosphatidylinositols. Tetrahedron Lett.

[41] Demel RA, Paltauf F, Hauser H (1987). Monolayer characteristics and thermal behavior of natural and synthetic phosphatidylserines. Biochemistry.

[42] Wang X, Devaiah SP, Zhang W, Welti R (2006). Signaling functions of phosphatidic acid. Prog Lipid Res.

[43] Kiyasu JY, Pieringer RA, Paulus H, Kennedy EP (1963). The biosynthesis of phosphatidylglycerol. J Biol Chem.

[44] Schlame M, Greenberg ML (1997). Cardiolipin synthase from yeast. BiochimBiophys Acta.

[45] Tripathi G, Chaurasiya K, Katare P (2013). Liposomal current status, evaluation and recent advances. Int J Curr Pharm Res.

[46] Ashara KC, Paun JS, Soniwala MM, Chavda JR, Nathawani SV, Mori NM (2014). Vesicular drug delivery system: a novel approach. Mintage J Pharm Med Sci.

[47] Lowry GV, Hill RJ, Harper S, Rawle AF, Hendren CO, Klaessig F (2016). Guidance to improve the scientific value of zeta-potential measurements in nanoEHS. Environ Sci Nano.

[48] Smith MC, Crist RM, Clogston JD, McNeil SE (2017). Zeta potential: a case study of cationic, anionic, and neutral liposomes. Anal Bioanal Chem.

[49] Rasmussen MK, Pedersen JN, Marie R (2020). Size and surface charge characterization of nanoparticles with a salt gradient. Nat Commun.

[50] Samad A, Sultana Y, Aqil M (2007). Liposomal drug delivery systems: an update review. Curr Drug Deliv.

[51] Jaafar-Maalej C, Diab R, Andrieu V, Elaissari A, Fessi H (2010). Ethanol injection method for hydrophilic and lipophilic drug-loaded liposome preparation. J Liposome Res.

[52] Deamer D, Bangham AD (1976). Large volume liposomes by an ether vaporization method. BiochimBiophys Acta.

[53] Rodriguez N, Pincet F, Cribier S (2005). Giant vesicles formed by gentle hydration and electroformation: a comparison by fluorescence microscopy. Colloids Surf B Biointerfaces.

[54] Meure LA, Foster NR, Dehghani F (2008). Conventional and dense gas techniques for the production of liposomes: a review. AAPS PharmSciTech.

[55] Mozafari MR (2005). Liposomes: an overview of manufacturing techniques. Cell Mol Biol Lett.

[56] van Swaay D, deMello A (2013). Microfluidic methods for forming liposomes. Lab Chip.

[57] Lou G, Anderluzzi G, Woods S, Roberts CW, Perrie Y (2019). A novel microfluidic-based approach to formulate size-tuneable large unilamellar cationic liposomes: formulation, cellular uptake and biodistribution investigations. Eur J Pharm Biopharm.

[58] Shaklee PM, Semrau S, Malkus M, Kubick S, Dogterom M, Schmidt T (2010). Protein incorporation in giant lipid vesicles under physiological conditions. Chembiochem.

[59] Lin YC, Huang KS, Chiang JT, Yang CH, Lai TH (2006). Manipulating self-assembled phospholipid microtubes using microfluidic technology. Sens Actuators B Chem.

[60] Jahn A, Vreeland WN, Gaitan M, Locascio LE (2004). Controlled vesicle self-assembly in microfluidic channels with hydrodynamic focusing. J Am Chem Soc.

[61] Funakoshi K, Suzuki H, Takeuchi S (2007). Formation of giant lipid vesiclelike compartments from a planar lipid membrane by a pulsed jet flow. J Am Chem Soc.

[62] Shum HC, Lee D, Yoon I, Kodger T, Weitz DA (2008). Double emulsion templated monodisperse phospholipid vesicles. Langmuir.

[63] Sugiura S, Kuroiwa T, Kagota T, Nakajima M, Sato S, Mukataka S (2008). Novel method for obtaining homogeneous giant vesicles from a monodisperse water-in-oil emulsion prepared with a microfluidic device. Langmuir.

[64] Ota S, Yoshizawa S, Takeuchi S (2009). Microfluidic formation of monodisperse, cell-sized, and unilamellar vesicles. Angew Chem Int Ed Engl.

[65] Matosevic S, Paegel BM (2011). Stepwise synthesis of giant unilamellar vesicles on a microfluidic assembly line. J Am Chem Soc.

[66] Otake K, Imura T, Sakai H, Abe M (2001). Development of a new preparation method of liposomes using supercritical carbon dioxide. Langmuir.

[67] Magnan C, Badens E, Commenges N, Charbit G (2000). Soy lecithin micronization by precipitation with a compressed fluid antisolvent-influence of process parameters. J Supercrit Fluids.

[68] Lesoin L, Crampon C, Boutin O, Badens E (2011). Preparation of liposomes using the supercritical anti-solvent (SAS) process and comparison with a conventional method. J Supercrit Fluids.

[69] Wang T, Deng Y, Geng Y, Gao Z, Zou J, Wang Z (2006). Preparation of submicron unilamellar liposomes by freeze-drying double emulsions. BiochimBiophys Acta.

[70] Laouini A, Jaafar-Maalej C, Sfar S, Charcosset C, Fessi H (2011). Liposome preparation using a hollow fiber membrane contactor--application to spironolactone encapsulation. Int J Pharm.

[71] Yu DG, Branford-White C, Williams GR, Bligh SW, White K, Zhu LM (2011). Self-assembled liposomes from amphiphilic electrospun nanofibers. Soft Matter.

[72] Genç R, Ortiz M, O’Sullivan CK (2009). Curvature-tuned preparation of nanoliposomes. Langmuir.

[73] Mouritsen OG (2011). Lipids, curvature, and nano-medicine. Eur J Lipid Sci Technol.

[74] Wiggenhorn M. Scale-Up of Liposome Manufacturing: Combining High Pressure Liposome Extrusion with Drying Technologies. Cuvillier Verlag; 2008.

[75] Shah VM, Nguyen DX, Patel P, Cote B, Al-Fatease A, Pham Y (2019). Liposomes produced by microfluidics and extrusion: a comparison for scale-up purposes. Nanomedicine.

[76] Charcosset C, Juban A, Valour JP, Urbaniak S, Fessi H (2015). Preparation of liposomes at large scale using the ethanol injection method: effect of scale-up and injection devices. Chem Eng Res Des.

[77] Carugo D, Bottaro E, Owen J, Stride E, Nastruzzi C (2016). Liposome production by microfluidics: potential and limiting factors. Sci Rep.

[78] Penoy N, Grignard B, Evrard B, Piel G (2021). A supercritical fluid technology for liposome production and comparison with the film hydration method. Int J Pharm.

[79] Kube S, Hersch N, Naumovska E, Gensch T, Hendriks J, Franzen A (2017). Fusogenic Liposomes as Nanocarriers for the Delivery of Intracellular Proteins. Langmuir.

[80] Madni A, Sarfraz M, Rehman M, Ahmad M, Akhtar N, Ahmad S (2014). Liposomal drug delivery: a versatile platform for challenging clinical applications. J Pharm Pharm Sci.

[81] Kaneda Y (2000). Virosomes: evolution of the liposome as a targeted drug delivery system. Adv Drug Deliv Rev.

[82] Scriboni AB, Couto VM, Ribeiro LNM, Freires IA, Groppo FC, de Paula E (2019). Fusogenic liposomes increase the antimicrobial activity of vancomycin against Staphylococcus aureus biofilm. Front Pharmacol.

[83] Csiszár A, Hersch N, Dieluweit S, Biehl R, Merkel R, Hoffmann B (2010). Novel fusogenic liposomes for fluorescent cell labeling and membrane modification. Bioconjug Chem.

[84] Yoshikawa T, Okada N, Nakagawa S (2006). Fusogenic liposomes and their suitability for gene delivery. Future Lipidol.

[85] Kunisawa J, Masuda T, Katayama K, Yoshikawa T, Tsutsumi Y, Akashi M (2005). Fusogenic liposome delivers encapsulated nanoparticles for cytosolic controlled gene release. J Control Release.

[86] Paliwal SR, Paliwal R, Vyas SP (2015). A review of mechanistic insight and application of pH-sensitive liposomes in drug delivery. Drug Deliv.

[87] Chen Y, Du Q, Guo Q, Huang J, Liu L, Shen X (2019). A W/O emulsion mediated film dispersion method for curcumin encapsulated pH-sensitive liposomes in the colon tumor treatment. Drug Dev Ind Pharm.

[88] Rehman AU, Omran Z, Anton H, Mély Y, Akram S, Vandamme TF (2018). Development of doxorubicin hydrochloride loaded pH-sensitive liposomes: investigation on the impact of chemical nature of lipids and liposome composition on pH-sensitivity. Eur J Pharm Biopharm.

[89] Liu X, Huang G (2013). Formation strategies, mechanism of intracellular delivery and potential clinical applications of pH-sensitive liposomes. Asian J Pharm Sci.

[90] Karanth H, Murthy RS (2007). pH-sensitive liposomes--principle and application in cancer therapy. J Pharm Pharmacol.

[91] Rayamajhi S, Marchitto J, Nguyen TDT, Marasini R, Celia C, Aryal S (2020). pH-responsive cationic liposome for endosomal escape mediated drug delivery. Colloids Surf B Biointerfaces.

[92] Monteiro LOF, Malachias Â, Pound-Lana G, Magalhães-Paniago R, Mosqueira VCF, Oliveira MC (2018). Paclitaxel-loaded pH-sensitive liposome: new insights on structural and physicochemical characterization. Langmuir.

[93] Kanamala M, Palmer BD, Jamieson SM, Wilson WR, Wu Z (2019). Dual pH-sensitive liposomes with low pH-triggered sheddable PEG for enhanced tumor-targeted drug delivery. Nanomedicine (Lond).

[94] Zou Y, Zong G, Ling YH, Perez-Soler R (2000). Development of cationic liposome formulations for intratracheal gene therapy of early lung cancer. Cancer Gene Ther.

[95] Heuts J, Varypataki EM, van der Maaden K, Romeijn S, Drijfhout JW, van Scheltinga AT (2018). Cationic liposomes: a flexible vaccine delivery system for physicochemically diverse antigenic peptides. Pharm Res.

[96] Shim G, Kim MG, Park JY, Oh YK (2013). Application of cationic liposomes for delivery of nucleic acids. Asian J Pharm Sci.

[97] Simões S, Filipe A, Faneca H, Mano M, Penacho N, Düzgünes N (2005). Cationic liposomes for gene delivery. Expert Opin Drug Deliv.

[98] Sharma A, Sharma US (1997). Liposomes in drug delivery: progress and limitations. Int J Pharm.

[99] Wrobel I, Collins D (1995). Fusion of cationic liposomes with mammalian cells occurs after endocytosis. BiochimBiophys Acta.

[100] Needham D, Dewhirst MW (2001). The development and testing of a new temperature-sensitive drug delivery system for the treatment of solid tumors. Adv Drug Deliv Rev.

[101] Wagner A, Vorauer-Uhl K (2011). Liposome technology for industrial purposes. J Drug Deliv.

[102] Rossmann C, McCrackin MA, Armeson KE, Haemmerich D (2017). Temperature sensitive liposomes combined with thermal ablation: effects of duration and timing of heating in mathematical models and in vivo. PLoS One.

[103] Lyu Y, Xiao Q, Yin L, Yang L, He W (2019). Potent delivery of an MMP inhibitor to the tumor microenvironment with thermosensitive liposomes for the suppression of metastasis and angiogenesis. Signal Transduct Target Ther.

[104] Kono K, Ozawa T, Yoshida T, Ozaki F, Ishizaka Y, Maruyama K (2010). Highly temperature-sensitive liposomes based on a thermosensitive block copolymer for tumor-specific chemotherapy. Biomaterials.

[105] van Elk M, Deckers R, Oerlemans C, Shi Y, Storm G, Vermonden T (2014). Triggered release of doxorubicin from temperature-sensitive poly(N-(2-hydroxypropyl)-methacrylamide mono/dilactate) grafted liposomes. Biomacromolecules.

[106] van Elk M, Ozbakir B, Barten-Rijbroek AD, Storm G, Nijsen F, Hennink WE (2015). Alginate microspheres containing temperature sensitive liposomes (TSL) for MR-guided embolization and triggered release of doxorubicin. PLoS One.

[107] Kneidl B, Peller M, Winter G, Lindner LH, Hossann M (2014). Thermosensitive liposomal drug delivery systems: state of the art review. Int J Nanomedicine.

[108] Lindner LH, Eichhorn ME, Eibl H, Teichert N, Schmitt-Sody M, Issels RD (2004). Novel temperature-sensitive liposomes with prolonged circulation time. Clin Cancer Res.

[109] Kataria S, Sandhu P, Bilandi A, Akanksha M, Kapoor B (2011). Stealth liposomes: a review. Int J Res Ayurveda Pharm.

[110] Pereira DSM, Cardoso BD, Rodrigues ARO, Amorim CO, Amaral VS, Almeida BG (2019). Magnetoliposomes containing calcium ferrite nanoparticles for applications in breast cancer therapy. Pharmaceutics.

[111] Cardoso BD, Rio ISR, Rodrigues ARO, Fernandes FCT, Almeida BG, Pires A (2018). Magnetoliposomes containing magnesium ferrite nanoparticles as nanocarriers for the model drug curcumin. R Soc Open Sci.

[112] Lorente C, Cabeza L, Clares B, Ortiz R, Halbaut L, Delgado Á V (2018). Formulation and in vitro evaluation of magnetoliposomes as a potential nanotool in colorectal cancer therapy. Colloids Surf B Biointerfaces.

[113] Bakandritsos A, Fatourou AG, Fatouros DG (2012). Magnetoliposomes and their potential in the intelligent drug-delivery field. TherDeliv.

[114] Joniec A, Sek S, Krysinski P (2016). Magnetoliposomes as potential carriers of doxorubicin to tumours. Chemistry.

[115] da Costa e Silva RM, Lara LR, López JL, Andrade ÂL, Oliveira JA, Takahashi JA (2018). Preparation of magnetoliposomes with a green, low-cost, fast and scalable methodology and activity study against S. aureus and C freundii bacterial strains. J Braz Chem Soc.

[116] Nappini S, Bombelli FB, Bonini M, Nordèn B, Baglioni P (2010). Magnetoliposomes for controlled drug release in the presence of low-frequency magnetic field. Soft Matter.

[117] Sabaté R, Barnadas-Rodríguez R, Callejas-Fernández J, Hidalgo-Alvarez R, Estelrich J (2008). Preparation and characterization of extruded magnetoliposomes. Int J Pharm.

[118] Choi WI, Sahu A, Wurm FR, Jo SM (2019). Magnetoliposomes with size controllable insertion of magnetic nanoparticles for efficient targeting of cancer cells. RSC Adv.

[119] Szuplewska A, Rękorajska Joniec A, Pocztańska E, Krysiński P, Dybko A, Chudy M (2019). Magnetic field-assisted selective delivery of doxorubicin to cancer cells using magnetoliposomes as drug nanocarriers. Nanotechnology.

[120] Shirmardi Shaghasemi B, Virk MM, Reimhult E (2017). Optimization of magneto-thermally controlled release kinetics by tuning of magnetoliposome composition and structure. Sci Rep.

[121] Monnier CA, Burnand D, Rothen-Rutishauser B, Lattuada M, Petri-Fink A (2014). Magnetoliposomes: opportunities and challenges. Eur J Nanomed.

[122] Yavlovich A, Smith B, Gupta K, Blumenthal R, Puri A (2010). Light-sensitive lipid-based nanoparticles for drug delivery: design principles and future considerations for biological applications. Mol Membr Biol.

[123] Puri A (2013). Phototriggerable liposomes: current research and future perspectives. Pharmaceutics.

[124] Miranda D, Lovell JF (2016). Mechanisms of light-induced liposome permeabilization. BioengTransl Med.

[125] Yavlovich A, Singh A, Blumenthal R, Puri A (2011). A novel class of photo-triggerable liposomes containing DPPC:DC(8,9)PC as vehicles for delivery of doxorubcin to cells. BiochimBiophys Acta.

[126] Juarranz Á, Jaén P, Sanz-Rodríguez F, Cuevas J, González S (2008). Photodynamic therapy of cancer Basic principles and applications. Clin Transl Oncol.

[127] Paasonen L, Sipilä T, Subrizi A, Laurinmäki P, Butcher SJ, Rappolt M (2010). Gold-embedded photosensitive liposomes for drug delivery: triggering mechanism and intracellular release. J Control Release.

[128] Danhier F, Feron O, Préat V (2010). To exploit the tumor microenvironment: passive and active tumor targeting of nanocarriers for anti-cancer drug delivery. J Control Release.

[129] Iyer AK, Khaled G, Fang J, Maeda H (2006). Exploiting the enhanced permeability and retention effect for tumor targeting. Drug Discov Today.

[130] Alavi M, Hamidi M. Passive and active targeting in cancer therapy by liposomes and lipid nanoparticles. Drug Metab Pers Ther 2019;34(1). 10.1515/dmpt-2018-0032. 30707682

[131] Maeda H, Bharate GY, Daruwalla J (2009). Polymeric drugs for efficient tumor-targeted drug delivery based on EPR-effect. Eur J Pharm Biopharm.

[132] Malam Y, Loizidou M, Seifalian AM (2009). Liposomes and nanoparticles: nanosized vehicles for drug delivery in cancer. Trends Pharmacol Sci.

[133] Okumura M, Ichihara H, Matsumoto Y (2018). Hybrid liposomes showing enhanced accumulation in tumors as theranostic agents in the orthotopic graft model mouse of colorectal cancer. Drug Deliv.

[134] Wu G, Li J, Yue J, Zhang S, Yunusi K (2018). Liposome encapsulated luteolin showed enhanced antitumor efficacy to colorectal carcinoma. Mol Med Rep.

[135] Zhang Y, Sriraman SK, Kenny HA, Luther E, Torchilin V, Lengyel E (2016). Reversal of chemoresistance in ovarian cancer by co-delivery of a P-glycoprotein inhibitor and paclitaxel in a liposomal platform. Mol Cancer Ther.

[136] Iwamoto Y, Matsumoto Y, Ueoka R (2005). Induction of apoptosis of human lung carcinoma cells by hybrid liposomes containing polyoxyethylenedodecyl ether. Int J Pharm.

[137] Liu Y, Lu WL, Guo J, Du J, Li T, Wu JW (2008). A potential target associated with both cancer and cancer stem cells: a combination therapy for eradication of breast cancer using vinorelbine stealthy liposomes plus parthenolide stealthy liposomes. J Control Release.

[138] Guo L, Fan L, Ren J, Pang Z, Ren Y, Li J (2012). Combination of TRAIL and actinomycin D liposomes enhances antitumor effect in non-small cell lung cancer. Int J Nanomedicine.

[139] Li X, Ding L, Xu Y, Wang Y, Ping Q (2009). Targeted delivery of doxorubicin using stealth liposomes modified with transferrin. Int J Pharm.

[140] Baek SE, Lee KH, Park YS, Oh DK, Oh S, Kim KS (2014). RNA aptamer-conjugated liposome as an efficient anticancer drug delivery vehicle targeting cancer cells in vivo. J Control Release.

[141] Bae YH, Park K (2011). Targeted drug delivery to tumors: myths, reality and possibility. J Control Release.

[142] Alshaer W, Hillaireau H, Vergnaud J, Ismail S, Fattal E (2015). Functionalizing liposomes with anti-CD44 aptamer for selective targeting of cancer cells. Bioconjug Chem.

[143] Liao ZX, Chuang EY, Lin CC, Ho YC, Lin KJ, Cheng PY (2015). An AS1411 aptamer-conjugated liposomal system containing a bubble-generating agent for tumor-specific chemotherapy that overcomes multidrug resistance. J Control Release.

[144] Byrne JD, Betancourt T, Brannon-Peppas L (2008). Active targeting schemes for nanoparticle systems in cancer therapeutics. Adv Drug Deliv Rev.

[145] Lian T, Ho RJ (2001). Trends and developments in liposome drug delivery systems. J Pharm Sci.

[146] Ye J, Yang Y, Jin J, Ji M, Gao Y, Feng Y (2020). Targeted delivery of chlorogenic acid by mannosylated liposomes to effectively promote the polarization of TAMs for the treatment of glioblastoma. Bioact Mater.

[147] Vakhshiteh F, Khabazian E, Atyabi F, Ostad SN, Madjd Z, Dinarvand R (2020). Peptide-conjugated liposomes for targeted miR-34a delivery to suppress breast cancer and cancer stem-like population. J Drug Deliv Sci Technol.

[148] Wang Z, Chi D, Wu X, Wang Y, Lin X, Xu Z (2019). Tyrosine modified irinotecan-loaded liposomes capable of simultaneously targeting LAT1 and ATB(0, + ) for efficient tumor therapy. J Control Release.

[149] Yang Y, Zhao Z, Xie C, Zhao Y (2020). Dual-targeting liposome modified by glutamic hexapeptide and folic acid for bone metastatic breast cancer. Chem Phys Lipids.

[150] Khayrani AC, Mahmud H, Oo AKK, Zahra MH, Oze M, Du J (2019). Targeting ovarian cancer cells overexpressing CD44 with immunoliposomes encapsulating glycosylated paclitaxel. Int J Mol Sci.

[151] Lin YL, Tsai NM, Chen CH, Liu YK, Lee CJ, Chan YL (2019). Specific drug delivery efficiently induced human breast tumor regression using a lipoplex by non-covalent association with anti-tumor antibodies. J Nanobiotechnology.

[152] Wöll S, Dickgiesser S, Rasche N, Schiller S, Scherließ R (2019). Sortagged anti-EGFR immunoliposomes exhibit increased cytotoxicity on target cells. Eur J Pharm Biopharm.

[153] Dumont N, Merrigan S, Turpin J, Lavoie C, Papavasiliou V, Geretti E (2019). Nanoliposome targeting in breast cancer is influenced by the tumor microenvironment. Nanomedicine.

[154] Nassir AM, Ibrahim IAA, Md S, Waris M, Tanuja Tanuja, Ain MR (2019). Surface functionalized folate targeted oleuropein nano-liposomes for prostate tumor targeting: in vitro and in vivo activity. Life Sci.

[155] Nguyen VD, Min HK, Kim CS, Han J, Park JO, Choi E (2019). Folate receptor-targeted liposomal nanocomplex for effective synergistic photothermal-chemotherapy of breast cancer in vivo. Colloids Surf B Biointerfaces.

[156] Yoon HY, Chang IH, Goo YT, Kim CH, Kang TH, Kim SY (2019). Intravesical delivery of rapamycin via folate-modified liposomes dispersed in thermo-reversible hydrogel. Int J Nanomedicine.

[157] Akhtar A, Ghali L, Wang SX, Bell C, Li D, Wen X (2019). Optimisation of folate-mediated liposomal encapsulated arsenic trioxide for treating HPV-positive cervical cancer cells in vitro. Int J Mol Sci.

[158] Fu J, Li W, Xin X, Chen D, Hu H (2020). Transferrin-modified nanoliposome codelivery strategies for enhancing the cancer therapy. J Pharm Sci.

[159] Wang X, Zhao Y, Dong S, Lee RJ, Yang D, Zhang H (2019). Cell-penetrating peptide and transferrin co-modified liposomes for targeted therapy of glioma. Molecules.

[160] Sakpakdeejaroen I, Somani S, Laskar P, Mullin M, Dufès C (2019). Transferrin-bearing liposomes entrapping plumbagin for targeted cancer therapy. J InterdiscipNanomed.

[161] Jhaveri A, Deshpande P, Pattni B, Torchilin V (2018). Transferrin-targeted, resveratrol-loaded liposomes for the treatment of glioblastoma. J Control Release.

[162] Bandekar A, Zhu C, Jindal R, Bruchertseifer F, Morgenstern A, Sofou S (2014). Anti-prostate-specific membrane antigen liposomes loaded with 225Ac for potential targeted antivascular α-particle therapy of cancer. J Nucl Med.

[163] Gui K, Zhang X, Chen F, Ge Z, Zhang S, Qi X (2019). Lipid-polymer nanoparticles with CD133 aptamers for targeted delivery of all-trans retinoic acid to osteosarcoma initiating cells. Biomed Pharmacother.

[164] Zhao Y, Xu J, Le VM, Gong Q, Li S, Gao F (2019). EpCAM aptamer-functionalized cationic liposome-based nanoparticles loaded with miR-139-5p for targeted therapy in colorectal cancer. Mol Pharm.

[165] Yu S, Bi X, Yang L, Wu S, Yu Y, Jiang B (2019). Co-delivery of paclitaxel and PLK1-targeted siRNA using aptamer-functionalized cationic liposome for synergistic anti-breast cancer effects in vivo. J Biomed Nanotechnol.

[166] Kulkarni SB, Betageri GV, Singh M (1995). Factors affecting microencapsulation of drugs in liposomes. J Microencapsul.

[167] Wang Y, Tu S, Pinchuk AN, Xiong MP (2013). Active drug encapsulation and release kinetics from hydrogel-in-liposome nanoparticles. J Colloid Interface Sci.

[168] Alavi M, Karimi N, Safaei M (2017). Application of various types of liposomes in drug delivery systems. Adv Pharm Bull.

[169] Saraf S, Jain A, Hurkat P, Jain SK (2016). Topotecan liposomes: a visit from a molecular to a therapeutic platform. Crit Rev Ther Drug Carrier Syst.

[170] Petrilli R, Eloy JO, Saggioro FP, Chesca DL, de Souza MC, Dias MVS (2018). Skin cancer treatment effectiveness is improved by iontophoresis of EGFR-targeted liposomes containing 5-FU compared with subcutaneous injection. J Control Release.

[171] Cullis PR, Mayer LD, Bally MB, Madden TD, Hope MJ (1989). Generating and loading of liposomal systems for drug-delivery applications. Adv Drug Deliv Rev.

[172] Brusa P, Immordino ML, Rocco F, Cattel L (2007). Antitumor activity and pharmacokinetics of liposomes containing lipophilic gemcitabine prodrugs. Anticancer Res.

[173] Gubernator J (2011). Active methods of drug loading into liposomes: recent strategies for stable drug entrapment and increased in vivo activity. Expert Opin Drug Deliv.

[174] Odeh F, Nsairat H, Alshaer W, Alsotari S, Buqaien R, Ismail S (2019). Remote loading of curcumin-in-modified β-cyclodextrins into liposomes using a transmembrane pH gradient. RSC Adv.

[175] Sur S, Fries AC, Kinzler KW, Zhou S, Vogelstein B (2014). Remote loading of preencapsulated drugs into stealth liposomes. Proc Natl Acad Sci U S A.

[176] Bally MB, Mayer LD, Loughrey H, Redelmeier T, Madden TD, Wong K (1988). Dopamine accumulation in large unilamellar vesicle systems induced by transmembrane ion gradients. Chem Phys Lipids.

[177] Dos Santos N, Cox KA, McKenzie CA, van Baarda F, Gallagher RC, Karlsson G (2004). pH gradient loading of anthracyclines into cholesterol-free liposomes: enhancing drug loading rates through use of ethanol. BiochimBiophys Acta.

[178] Dos Santos N, Mayer LD, Abraham SA, Gallagher RC, Cox KA, Tardi PG (2002). Improved retention of idarubicin after intravenous injection obtained for cholesterol-free liposomes. BiochimBiophys Acta.

[179] Haran G, Cohen R, Bar LK, Barenholz Y (1993). Transmembrane ammonium sulfate gradients in liposomes produce efficient and stable entrapment of amphipathic weak bases. BiochimBiophys Acta.

[180] Wei H, Song J, Li H, Li Y, Zhu S, Zhou X (2013). Active loading liposomal irinotecan hydrochloride: preparation, in vitro and in vivo evaluation. Asian J Pharm Sci.

[181] Zucker D, Marcus D, Barenholz Y, Goldblum A (2009). Liposome drugs’ loading efficiency: a working model based on loading conditions and drug’s physicochemical properties. J Control Release.

[182] Clerc S, Barenholz Y (1995). Loading of amphipathic weak acids into liposomes in response to transmembrane calcium acetate gradients. BiochimBiophys Acta.

[183] Chen H, MacDonald RC, Li S, Krett NL, Rosen ST, O’Halloran TV (2006). Lipid encapsulation of arsenic trioxide attenuates cytotoxicity and allows for controlled anticancer drug release. J Am Chem Soc.

[184] Deamer DW, Prince RC, Crofts AR (1972). The response of fluorescent amines to pH gradients across liposome membranes. BiochimBiophys Acta.

[185] Fenske DB, Wong KF, Maurer E, Maurer N, Leenhouts JM, Boman N (1998). Ionophore-mediateduptake of ciprofloxacin and vincristine into large unilamellar vesicles exhibiting transmembrane ion gradients. BiochimBiophys Acta.

[186] Gubernator J, Chwastek G, Korycińska M, Stasiuk M, Grynkiewicz G, Lewrick F (2010). The encapsulation of idarubicin within liposomes using the novel EDTA ion gradient method ensures improved drug retention in vitro and in vivo. J Control Release.

[187] Fritze A, Hens F, Kimpfler A, Schubert R, Peschka-Süss R (2006). Remote loading of doxorubicin into liposomes driven by a transmembrane phosphate gradient. BiochimBiophys Acta.

[188] Tang WL, Tang WH, Szeitz A, Kulkarni J, Cullis P, Li SD (2018). Systemic study of solvent-assisted active loading of gambogic acid into liposomes and its formulation optimization for improved delivery. Biomaterials.

[189] Hwang H, Jeong HS, Oh PS, Kim M, Lee TK, Kwon J (2016). PEGylated nanoliposomes encapsulating angiogenic peptides improve perfusion defects: radionuclide imaging-based study. Nucl Med Biol.

[190] Lin C, Zheng H, Sun M, Guo Y, Luo F, Guo L (2018). Highly sensitive colorimetric aptasensor for ochratoxin A detection based on enzyme-encapsulated liposome. Anal Chim Acta.

[191] Chen Q, Shang W, Zeng C, Wang K, Liang X, Chi C (2017). Theranostic imaging of liver cancer using targeted optical/MRI dual-modal probes. Oncotarget.

[192] Silindir M, Erdoğan S, Özer AY, Maia S (2012). Liposomes and their applications in molecular imaging. J Drug Target.

[193] Zhang L, Gu FX, Chan JM, Wang AZ, Langer RS, Farokhzad OC (2008). Nanoparticles in medicine: therapeutic applications and developments. Clin PharmacolTher.

[194] Zylberberg C, Matosevic S (2016). Pharmaceutical liposomal drug delivery: a review of new delivery systems and a look at the regulatory landscape. Drug Deliv.

[195] Moghimipour E, Handali S (2013). Liposomes as drug delivery systems: properties and applications. Res J Pharm Biol Chem Sci.

[196] Sen R, Satpathy S (2014). Liposomes as drug delivery system: a brief review. Int J Res Pharm Sci.

[197] Abbasi H, Rahbar N, Kouchak M, Khalil Dezfuli P, Handali S (2022). Functionalized liposomes as drug nanocarriers for active targeted cancer therapy: a systematic review. J Liposome Res.

[198] Cedervall T, Lynch I, Foy M, Berggård T, Donnelly SC, Cagney G (2007). Detailed identification of plasma proteins adsorbed on copolymer nanoparticles. Angew Chem Int Ed Engl.

[199] Mustafa Khidir A, Saeed AA (2020). Ligand-targeted liposomes. Health Prim Care.

[200] Lundqvist M, Stigler J, Elia G, Lynch I, Cedervall T, Dawson KA (2008). Nanoparticle size and surface properties determine the protein corona with possible implications for biological impacts. Proc Natl Acad Sci U S A.

[201] Lammers T, Kiessling F, Hennink WE, Storm G (2012). Drug targeting to tumors: principles, pitfalls and (pre-) clinical progress. J Control Release.

[202] Fathi S, Oyelere AK (2016). Liposomal drug delivery systems for targeted cancer therapy: is active targeting the best choice?. Future Med Chem.

[203] Dwivedi C, Verma S (2013). Review on preparation and characterization of liposomes with application. Int J Sci Innov Res.

[204] Zalba S, Garrido MJ (2013). Liposomes, a promising strategy for clinical application of platinum derivatives. Expert Opin Drug Deliv.

