# Supplementary material for: What We Need to Know about Liposomes as Drug Nanocarriers: An Updated Review
Source: Adv Pharm Bull. 2022 Apr 4;13(1):7–23. doi: 10.34172/apb.2023.009 (PMC9871273; doi:10.34172/apb.2023.009)
Supplement: Supplementary file 1 — contains Figures S1-S5. [file apb-13-7-s001.pdf]

Adv Pharm Bull, 2023, 13(1), 7-23
doi: 10.34172/apb.2023.009
https://apb.tbzmed.ac.irhttps://apb.tbzmed.ac.ir

What We Need to Know about Liposomes as Drug Nanocarriers: An Updated Review

Hanieh Abbasi[1,2], Maryam Kouchak[2,3], Zohreh Mirveis[1,2], Fatemeh Hajipour[1], Mohsen Khodarahmi[1,2], Nadereh Rahbar[2*], Somayeh Handali[4*]

[1]Department of Medicinal Chemistry, School of Pharmacy, Ahvaz Jundishapur University of Medical Sciences, Ahvaz, Iran.
[2]Nanotechnology Research Center, Ahvaz Jundishapur University of Medical Sciences, Ahvaz, Iran.
[3]Department of Pharmaceutics, School of Pharmacy, Ahvaz Jundishapur University of Medical Sciences, Ahvaz, Iran.
[4]Medical Biomaterials Research Center (MBRC), Tehran University of Medical Sciences, Tehran, Iran.

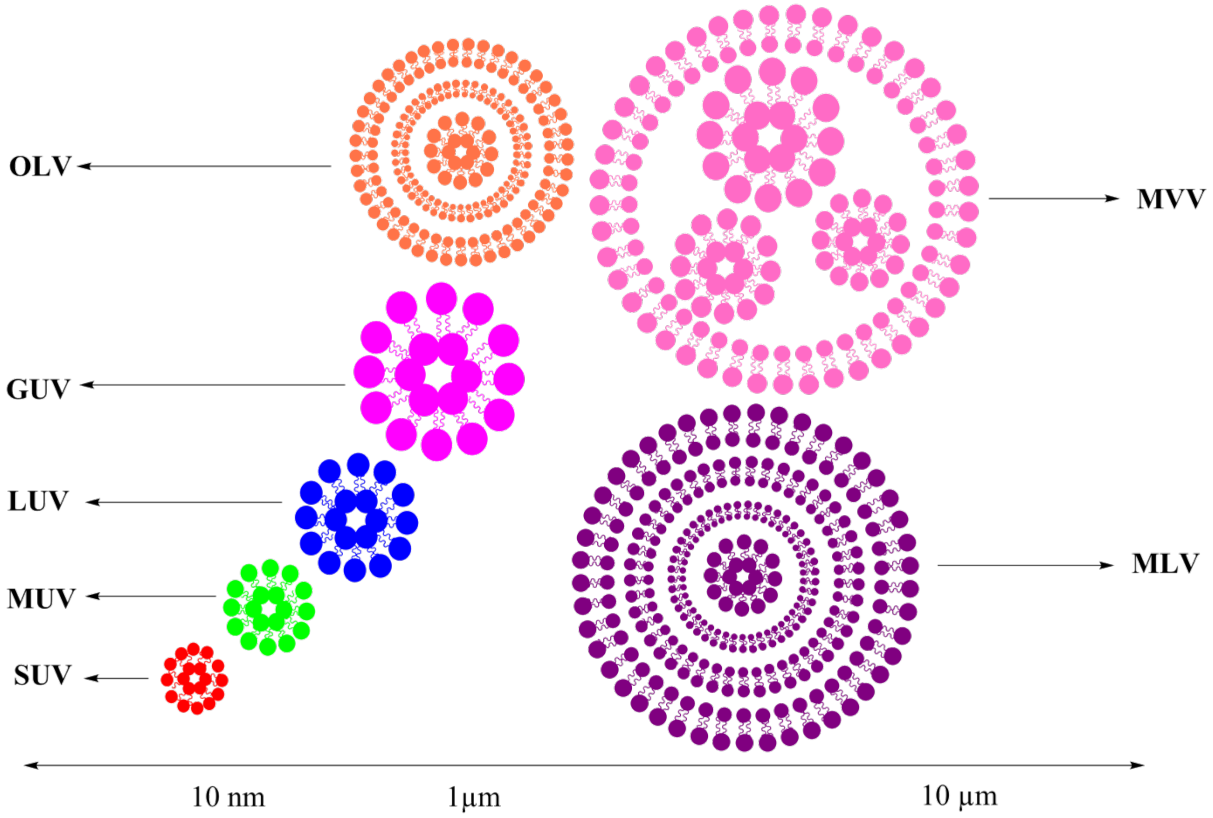

Figure S1. Classification of liposomes according to their size and the number of layers.

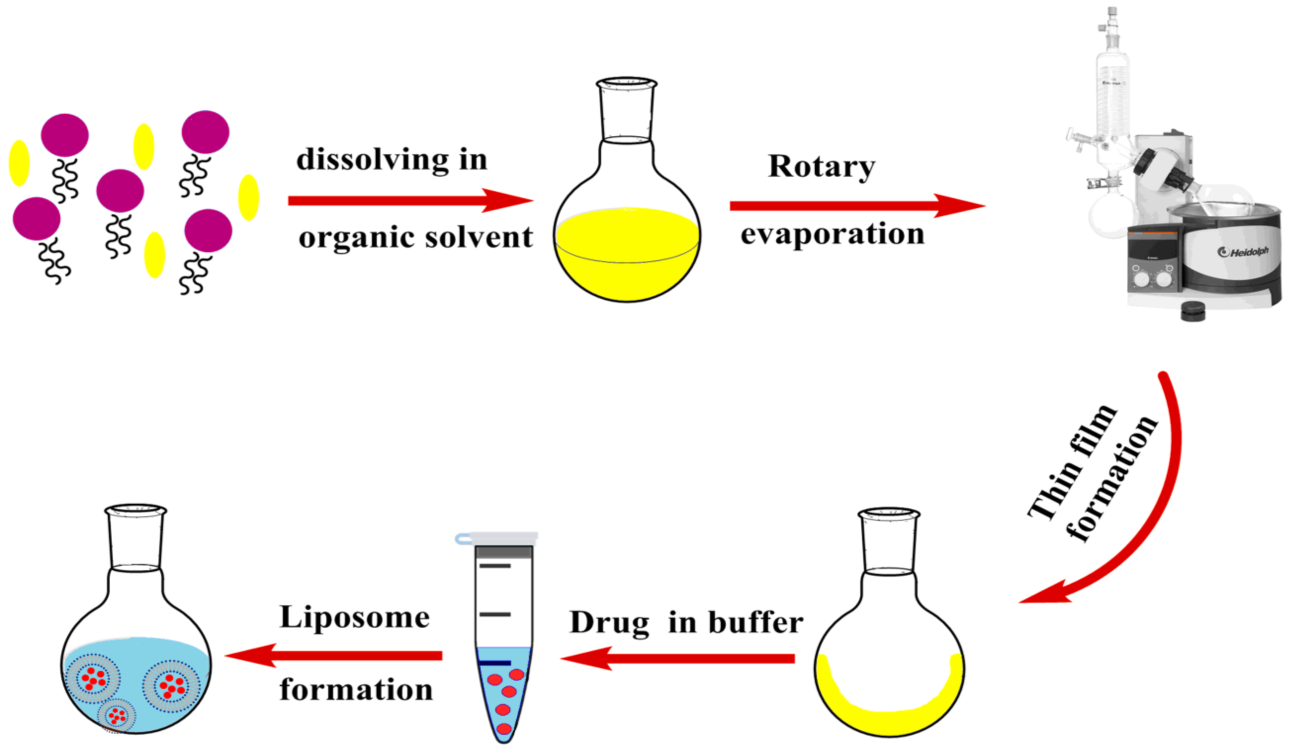

Figure S2. Schematic presentation of liposome preparation by “Thin Film” method.

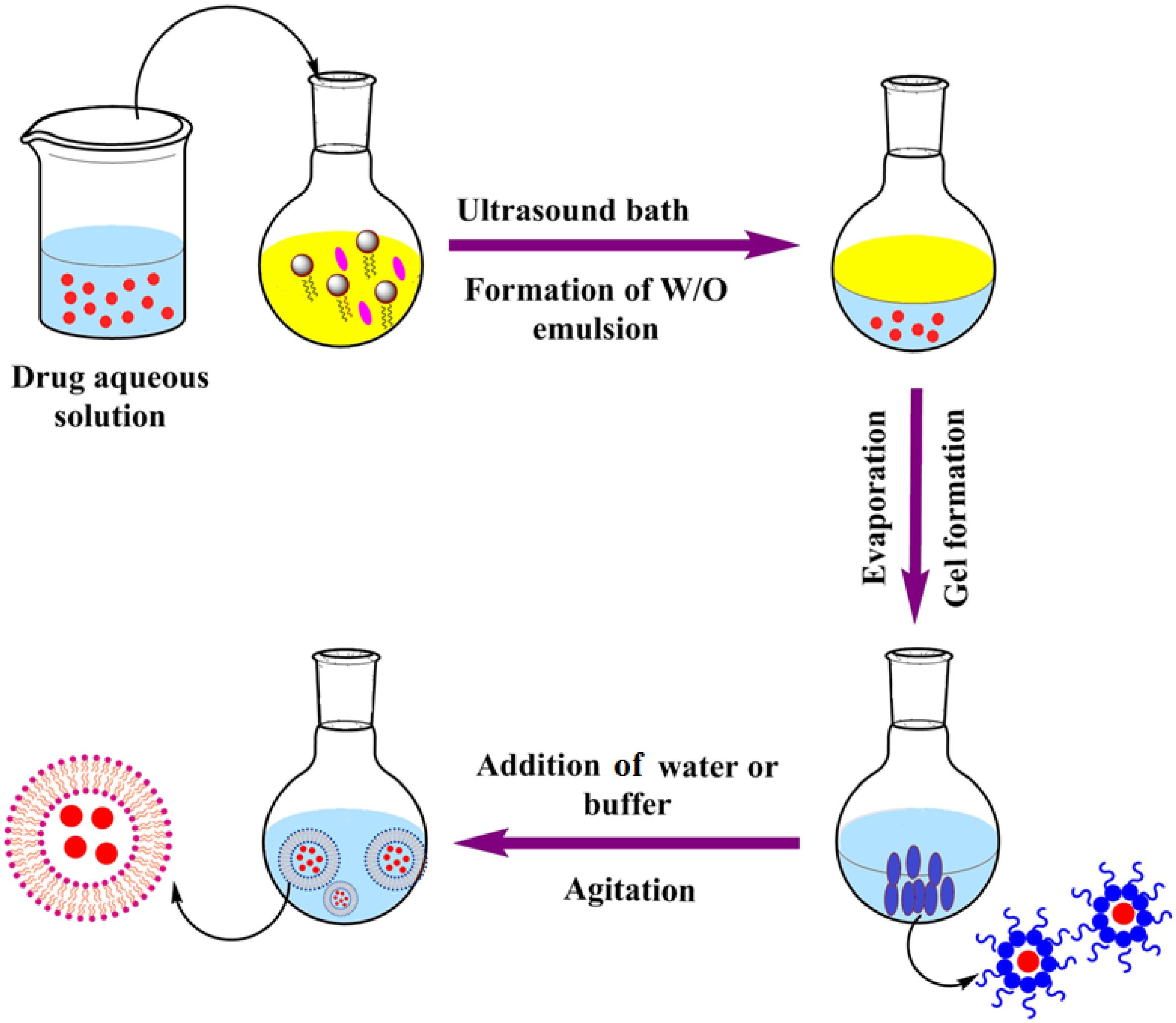

Figure S3. Schematic presentation of liposome preparation by “Reverse Phase Evaporation” method.

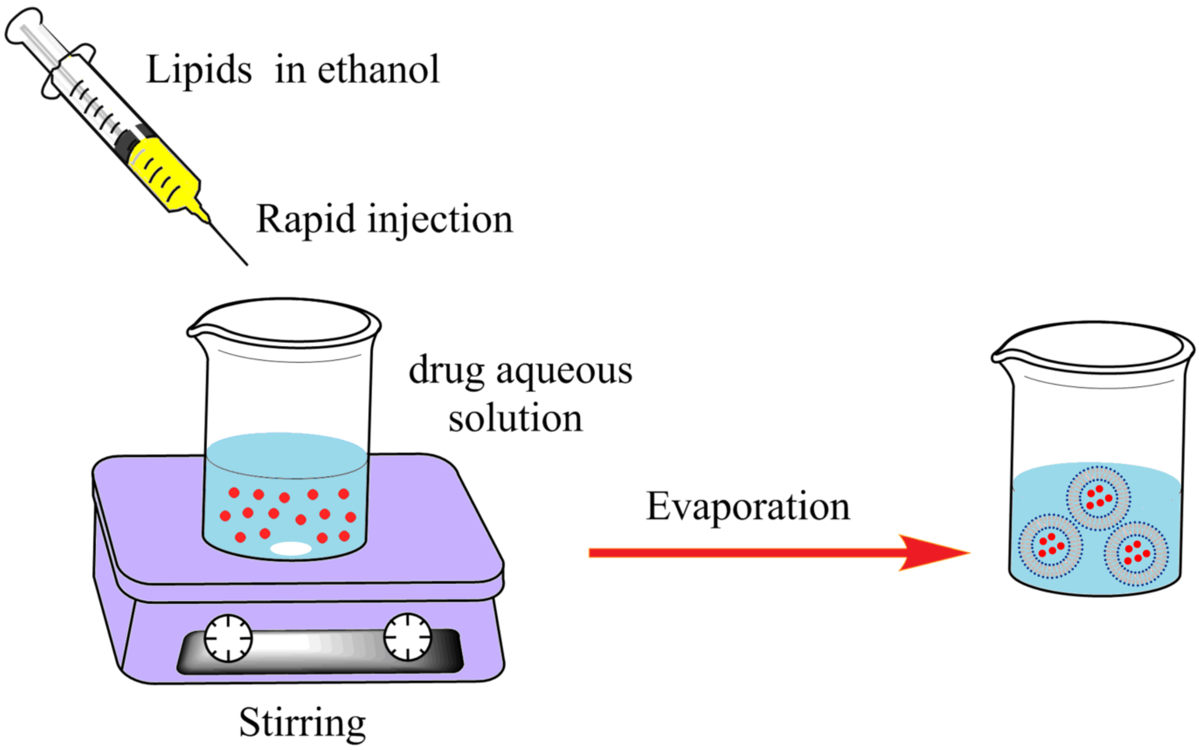

Figure S4. Schematic presentation of liposome preparation by “Ethanol Injection” method.

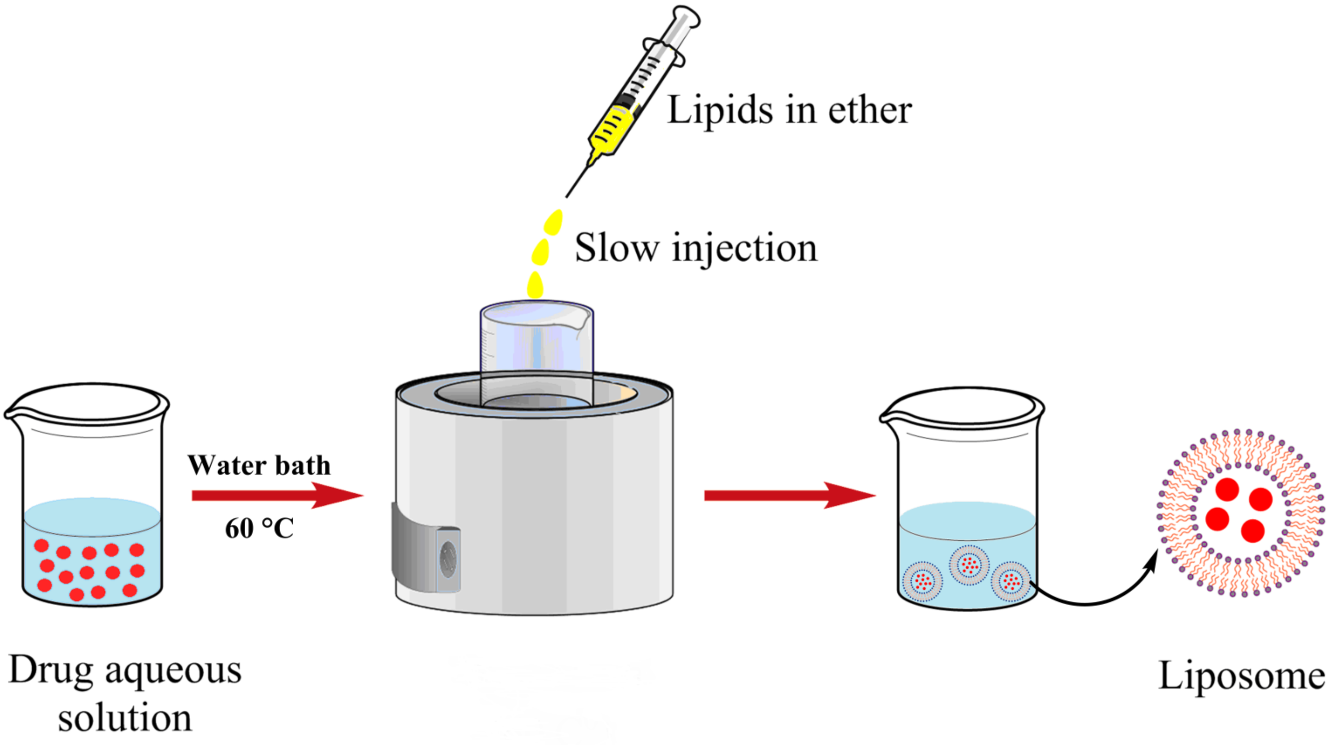

Figure S5. Schematic presentation of liposome preparation by “Ether Injection” (solvent vaporization) method.
